# Uterus globulin associated protein 1 (UGRP1) binds podoplanin (PDPN) to promote a novel inflammation pathway during *Streptococcus pneumoniae* infection

**DOI:** 10.1002/ctm2.850

**Published:** 2022-06-02

**Authors:** Lei Han, Feifei Zhang, Yu Liu, Jie Yu, Qianyue Zhang, Xiaoping Ye, Huaidong Song, Cuixia Zheng, Bing Han

**Affiliations:** ^1^ The Core Laboratory in Medical Center of Clinical Research, Department of Molecular Diagnostics and Endocrinology, Shanghai Ninth People's Hospital, State Key Laboratory of Medical Genomics Shanghai Jiao Tong University School of Medicine Shanghai China; ^2^ Department of Ophthalmology, Shanghai Ninth People's Hospital Shanghai Jiao Tong University School of Medicine Shanghai China; ^3^ Department of Respiration, Yangpu Hospital Tongji University School of Medicine Shanghai China; ^4^ Department of Endocrinology, Shanghai Ninth People's Hospital Shanghai Jiao Tong University School of Medicine Shanghai China

**Keywords:** UGRP1, PDPN, macrophage, IL‐6

## Abstract

**Background:**

*Streptococcus pneumoniae* is the major cause of life‐threatening infections. Toll‐like receptors (TLRs) and NOD‐like receptors (NLRs) could recognise *S. pneumoniae* and regulate the production of pro‐inflammatory cytokines. UGRP1, highly expressed in lung, is predominantly secreted in airways. However, the function of UGRP1 in pneumonia is mainly unknown.

**Methods and results:**

We showed that upon TLR2/TLR4/NOD2 agonists stimulation or *S. pneumoniae* infection, treatment with UGRP1 could promote phosphorylation of p65 and enhance IL‐6, IL‐1β and TNFα production in macrophages. We further elucidated that after binding with cell‐surface receptor PDPN, UGRP1 could activate RhoA to enhance interaction of IKKγ and IKKβ, which slightly activated NF‐κB to improve expression of TLR2, MyD88, NOD2 and NLRP3. Deletion of UGRP1 or blocking UGRP1 interaction with PDPN protected mice against *S. pneumoniae*‐induced severe pneumococcal pneumonia, and activating RhoA with agonist in UGRP1‐deficient mice restored the reduced IL‐6 production.

**Conclusion:**

We demonstrated that UGRP1–PDPN–RhoA signaling could activate NF‐κB to promote expression of TLR2, MyD88, NOD2 and NLRP3, which enhanced inflammatory cytokines secretion during *S. pneumoniae* infection. Antibodies, which could interrupt interaction of UGRP1 and PDPN, are potential therapeutics against *S. pneumoniae*.

## Graphical abstract

1. UGRP1–PDPN signalling activate RhoA to enhance the interaction of IKKγ and IKKβ.

2. Interaction of IKKγ and IKKβ activate NF‐κB to enhance expression of TLR2, MyD88 and NOD2.

3. Upon *S. pneumoniae* infection, increased expression of TLR2, MyD88 and NOD2 could generate enhanced NF‐κB signalling to produce more pro‐inflammatory cytokines including IL‐6, IL‐1β and TNFα.

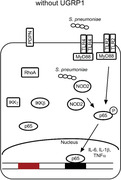

## 1 INTRODUCTION

As a gram‐positive bacterium, *Streptococcus pneumoniae* exists in and invades the respiratory tract. It is the major cause of community acquired pneumonia and results in about 90% pneumonia deaths, especially in young children and the elderly.^1^, ^2^ So, it is an important cause of morbidity and mortality worldwide.^2^

With pattern recognition receptors (PRRs) of the innate immune system, host could firstly recognised *S. pneumoniae*. Toll‐like receptors (TLRs) and NOD‐like receptors (NLRs) have been identified as different classes of PRRs. Bacterial virulence factors and conserved microbial molecules can activate these receptors.^3^, ^4^ After activation, most PPRs stimulate the transcription factor NF‐κB to produce the pro‐inflammatory cytokines, including IL‐6, IL‐1β and TNFα. Subsequently, these cytokines regulate the adaptive immunity by stimulating immune cells, activating the acute‐phase response and recruiting neutrophils and macrophages.^5^

The host could be protected from *S. pneumoniae* by balanced inflammation, while would be harmed by excessive inflammation induced by TLR and NLR signalling pathways extreme activation. The most dying patients with severe pneumococcal pneumonia are commonly caused by rigorous inflammation. Compared with other cytokines, IL‐6 shows more inflammatory effect and correlates prominently with death rates in the patients with severe pneumococcal pneumonia.^6^

Extracellular proteins have been reported to regulate TLR signalling pathway by more and more studies. It was reported that vascular endothelial growth factor C (VEGF‐C) and its cell surface receptor VEGFR‐3 could restrain TLR4‐NF‐κB signalling and IL‐6 expression.^7^ Tumor‐secreted protein S (Pros1), a Mer/Tyro3 ligand, was also demonstrated to decrease IL‐6 expression in LPS and IFNγ‐stimulated macrophages.^8^ Same as extracellular protein, deficiency of tissue inhibitor of metalloproteinases‐3 results in increased IL‐6 expression in LPS‐induced macrophages.^9^

Uterus globulin‐associated protein 1 (UGRP1), with the other name of SCGB3A2, is an extracellular protein mainly secreted by lung cells. The epithelial cells of the bronchial tubes, bronchus and trachea have the high expression of UGRP1, while thyroid gland also expresses UGRP1 lowly.^10^ It was reported that UGRP1 was involved in pathogenesis of autoimmune thyroid disease (AITD) and enhanced expression of UGRP1 was reported in thyroids of AITD patients.^11^, ^12^ Chiba et al.^13^ showed that UGRP1 can restrain inflammation in the mouse model of allergic airway inflammation. Other studies also showed UGRP1 had anti‐inflammatory property and recombinant human UGRP1 could be used as an anti‐inflammatory agent in allergic pulmonary inflammation.^14^, ^15^ However, the function of UGRP1 in inflammation is still controversial. It was reported that UGRP1 could combine LPS and deliver LPS to the cytosol, which activate caspase‐11 to induce maturation of IL‐1β and pyroptosis of macrophages.^16^, ^17^ Because of the relationship of UGRP1 and inflammation described above, we intended to investigate the function of UGRP1 in lung inflammation induced by *S. pneumoniae*.

In this study, we showed that UGRP1 positively regulated IL‐6, IL‐1β and TNFα production in *S. pneumoniae‐*infected or TLR2/TLR4/NOD2 agonists‐treated macrophages, which was dependent on the combination of UGRP1 and cell‐surface receptor PDPN (podoplanin). Furthermore, interaction of UGRP1 and PDPN activates RhoA to enhance interaction of IKKγ and IKKβ, which slightly activates NF‐κB to increase expression of TLR2, MyD88, NOD2 and NLRP3. We found a novel inflammation pathway that enhanced NF‐κB phosphorylation in an TLR2/4 independent manner.

## 2 RESULTS

### 2.1 UGRP1 was identified to positively regulate inflammation in *S. pneumoniae* stimulated macrophages

**FIGURE 1:**
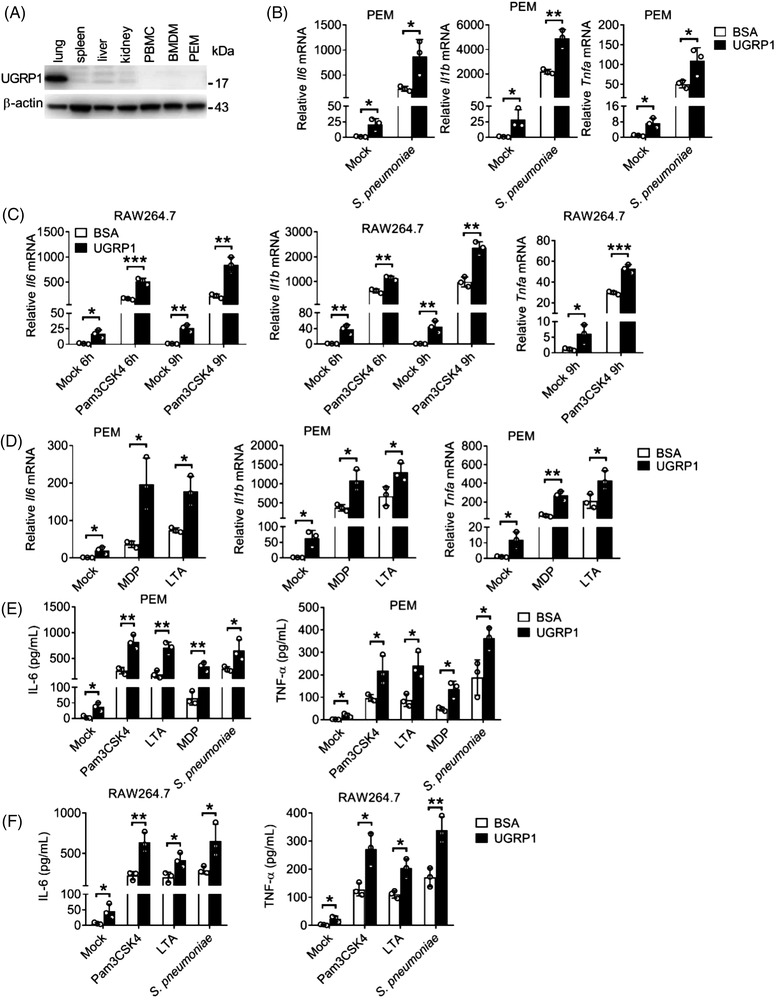
UGRP1 was identified to positively regulate inflammation in *S. pneumoniae* stimulated macrophages. (A) Immunoblot analysis of UGRP1 protein expression in murine lung, spleen, liver, kidney, PBMCs, BMDMs and PEMs. (B) PEMs were treated with BSA or UGRP1 (0.5 μg/ml) for 1 h followed by *S. pneumoniae* (10^4^ CFU/well) infection for 6 h to check *Il6, Il1b* and *Tnfa* mRNA by qRT‐PCR (*n* = 3). (C) RAW264.7 cells were treated with BSA or UGRP1 (0.5 μg/ml) for 1 h followed by Pam3CSK4 (0.5 μg/ml) stimulation for 6 or 9 h to check *Il6, Il1b* and *Tnfa* mRNA by qRT‐PCR (*n* = 3). (D) PEMs were treated with BSA or UGRP1 (0.5 μg/ml) for 1 h followed by MDP (1 μg/ml) or LTA (0.5 μg/ml) stimulation for 6h to check *Il6, Il1b* and *Tnfa* mRNA by qRT‐PCR (*n* = 3). (E and F) PEMs (E, *n* = 3) or RAW264.7 cells (F, *n* = 3) were treated with BSA or UGRP1 (0.5 μg/ml) for 1 h followed by Pam3CSK4 (0.5 μg/ml), LTA (0.5 μg/ml), MDP (1 μg/ml) stimulation or *S. pneumoniae* (10^4 ^CFU/well) infection for 12 h to check IL‐6 and TNFα concentrations by ELISA. *p < .05, **p < .01 and ***p < .001, using two‐way analysis of variance (ANOVA) with Holm–Sidak's multiple comparisons test (B–F). Data from at least three independent experiments (mean ± SD) or representative data (A). Please also see Figure S1

We first assessed UGRP1 expression in different murine organs and cells. Then, we found UGRP1 was highly expressed in lung and there was no UGRP1 expression in spleen, liver, kidney, peripheral blood mononuclear cells (PBMCs), bone marrow‐derived macrophages (BMDMs) and peritoneal exudate macrophages (PEMs) (Figure 1A and Figure S1A), which was identical to previous reports.^18^ To investigate whether UGRP1 participated in inflammation response after *S. pneumoniae* infection, we treated *S. pneumoniae*‐stimulated PEMs with UGRP1 and found UGRP1 increased mRNA levels of *Il6, Il1b* and *Tnfa* (Figure 1B). Meanwhile, we also found UGRP1 slightly increased mRNA levels of *Il6, Il1b* and *Tnfa* in PEMs without infection of *S. pneumoniae* (Figure 1B). With the consideration that some components of *S. pneumoniae* could be recognised by TLRs and NLRs including TLR2, TLR4 and NOD2,^19^ which was also verified by gene silencing studies (Figure S1B,C), we stimulated macrophages with Pam3CSK4 (agonist of TLR2), LTA (agonist of TLR2), LPS (agonist of TLR4) or MDP (agonist of NOD2) with or without UGRP1. In Pam3CSK4 or LPS stimulated RAW264.7 cells (murine macrophage cell lines), UGRP1 enhanced mRNA levels of *Il6* and *Il1b* in 6 and 9 h, simultaneously with enhanced *Tnfa* mRNA levels in 9 h (Figure 1C and Figure S1D). Consistently, with the stimulation of MDP or LTA, UGRP1 increased mRNA levels of *Il6, Il1b* and *Tnfa* in PEMs (Figure 1D). Also, in LPS stimulated PEMs and BMDMs, UGRP1 enhanced *Il6, Il1b* and *Tnfa* mRNA levels (Figure S1E,F). Then, we also confirmed UGRP1‐mediated increased expression of IL‐6 and TNFα by ELISA in Pam3CSK4, LTA, MDP, LPS or *S. pneumoniae* stimulated PEMs (Figure 1E and Figure S1G) and RAW 264.7 cells (Figure 1F and Figure S1H). In addition, we observed that UGRP1 could not enhance the anti‐inflammatory cytokine *Il10* mRNA in Pam3CSK4‐stimulated PEMs, although slightly increased *Il10* mRNA could be induced by UGRP1 alone (Figure S1I). Together, we have identified that the UGRP1 enhances TLR2/4 and NOD2‐induced inflammation in macrophages.

### 2.2 UGRP1 enhances TLR2‐induced NF‐κB activation

**FIGURE 2:**
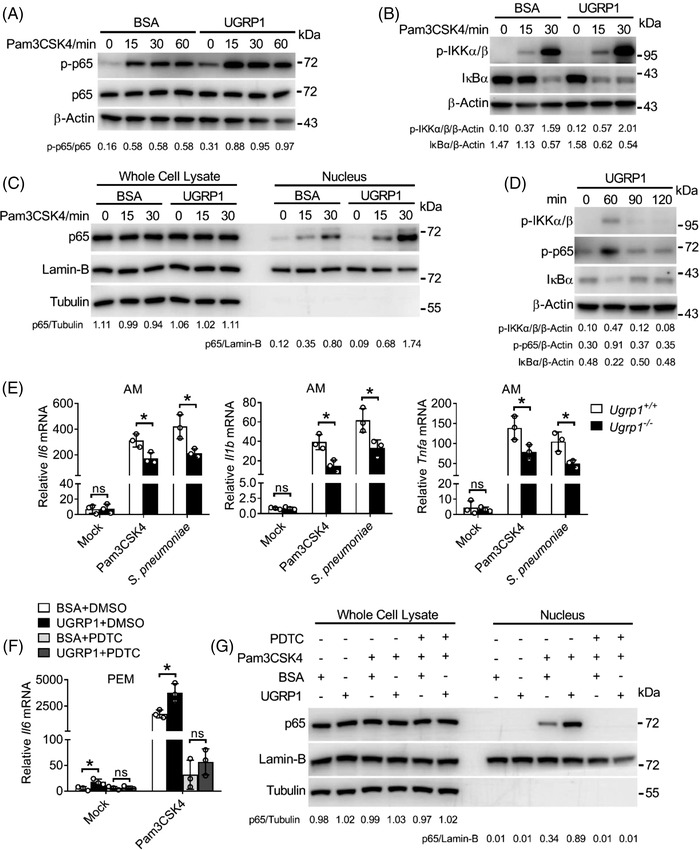
UGRP1 enhances TLR2‐induced NF‐κB activation. (A–C) Immunoblot analysis of phosphorylated (p‐) p65 (A), p‐IKKα/β, degradation of IκBα (B) and p65 in the whole cell lysate (C, left) and nucleus (C, right) in BSA or UGRP1‐treated PEMs for 6 h following Pam3CSK4 stimulation for the indicated periods. β‐Actin and tubulin were used as the whole lysate protein control, Lamin‐B served as the nucleic protein control. (D) Immunoblot analysis of p‐IKKα/β, p‐p65 and degradation of IκBα in PEMs with treatment of UGRP1 for the indicated periods. (E) Alveolar macrophages (AMs) from WT or UGRP1 KO mice were stimulated with Pam3CSK4 or *S. pneumoniae* for 6 h to check *Il6, Il1b* and *Tnfa* mRNA by qRT‐PCR (*n* = 3). (F) PEMs were treated with the NF‐κB inhibitor PDTC (100μM) for 1 h followed by BSA or UGRP1 (0.5 μg/ml) treatment and Pam3CSK4 stimulation for 6 h to measure *Il6* mRNA by qRT‐PCR (*n* = 3). (G) PEMs were treated with the NF‐κB inhibitor PDTC (100 μM) for 1h followed by BSA or UGRP1 (0.5 μg/ml) treatment for 6 h, then stimulated by Pam3CSK4 for 0.5 h, p65 in the whole cell lysate (left) and nucleus (right) was analysed by immunoblot. Tubulin were used as the whole lysate protein control, Lamin‐B served as the nucleic protein control. *p < .05, **p < .01 and ***p < .001, using two‐way analysis of variance (ANOVA) with Holm–Sidak's multiple comparisons test (E and F). Data from at least three independent experiments (mean ± SD) or representative data (A–D, G). Please also see Figure S2

As one of the main transcription factors activated by Pam3CSK4, NF‐κB can induce the expression of inflammatory cytokines. Following phosphorylation and activation of IKKα/β, IκBα is phosphorylated and polyubiquitinated for subsequent degradation, resulting in nuclear entry of NF‐κB.^20^ Therefore, we wonder whether or not UGRP1 could affect the NF‐κB signalling pathway. Consistent with our former data, UGRP1‐treated PEMs showed enhanced phosphorylation levels of p65 (subunit of NF‐κB) and IKKα/β with increased degradation of IκBα following Pam3CSK4 stimulation (Figure 2A,B). Simultaneously, enhanced translocation of p65 into nucleus was also observed in UGRP1‐treated PEMs (Figure 2C and Figure S2A). Interestingly, following UGRP1 treatment for 1 h without any other stimulation, PEMs showed slightly increased phosphorylation of p65 and IKKα/β and degradation of IκBα (Figure 2D). Furthermore, p65 phosphorylation and IκBα degradation after UGRP1 treatment were much weaker than those after Pam3CSK4 stimulation for 1 h (Figure S2B). These results were in accordance with slightly enhanced expression of *Il6, Il1b* and *Tnfa* in PEMs, BMDMs and RAW264.7 cells after UGRP1 treatment alone (Figure 1B–F and Figure S1D–H).

In order to better understand the function of UGRP1, we generated UGRP1 knockout (KO) mice with a recombineering‐based method. 3.6 kbp genomic sequence including the whole *Ugrp1* gene was replaced by 2.3 kbp DNA fragment containing Neomycin cassette (Figure S2C). The efficiency of UGRP1 KO in lungs was confirmed by western blotting (Figure S2D). Normal percentages of B220^+^, CD4^+^ and CD8^+^ lymphocytes, and F4/80^+^ CD11b^+^ macrophages in spleen were demonstrated in UGRP1 KO mice, while normal T cell development in the thymus were also showed in UGRP1 KO mice, which were bred in a specific‐pathogen‐free facility (Figure S2E). Bone marrow cells from UGRP1 KO mice were then induced with M‐CSF, and the development of BMDMs were not affected, showed normal percentages of F4/80^+^CD11b^+^ macrophages (Figure S2F). Then, we observed reduced mRNA levels of *Il6, Il1b* and *Tnfa* in alveolar macrophages (AMs; Figure 2E), but unchanged in PEMs and BMDMs (Figure S2G,H) from UGRP1 KO mice after treated with Pam3CSK4 or *S. pneumoniae*, which would be explained by positive expression of UGRP1 in lung but negative expression of UGRP1 in PEMs, BMDMs and any other cells we tested (Figure 1A and Figure S1A). These results were in accordance with average 50–100 times of UGRP1's concentration in BAL fluid than in serum,^21^ indicating that UGRP1 mainly play a role in lung. Then, during stimulation of Pam3CSK4, adding back UGRP1 protein restored the reduced *Il6* and *Tnfa* mRNA levels in AMs derived from UGRP1‐deficient mice (Figure S2I). To determine whether NF‐κB is the central transcription factor downstream of UGRP1, we next blocked NF‐κB activation by pyrimidine‐dithiocarbamate (PDTC) treatment, which profoundly suppressed *Il6* mRNA (Figure 2F) and nucleus translocated p65 (Figure 2G) to the similar levels in UGRP1‐treated and ‐untreated PEMs followed by Pam3CSK4 stimulation. Collectively, we have demonstrated that UGRP1 enhances *S. pneumoniae*‐induced NF‐κB activation and pro‐inflammatory cytokine production in macrophages.

### 2.3 UGRP1 enhances expression of TLR2, NOD2, MyD88 and NLRP3 by activation of NF‐κB

**FIGURE 3:**
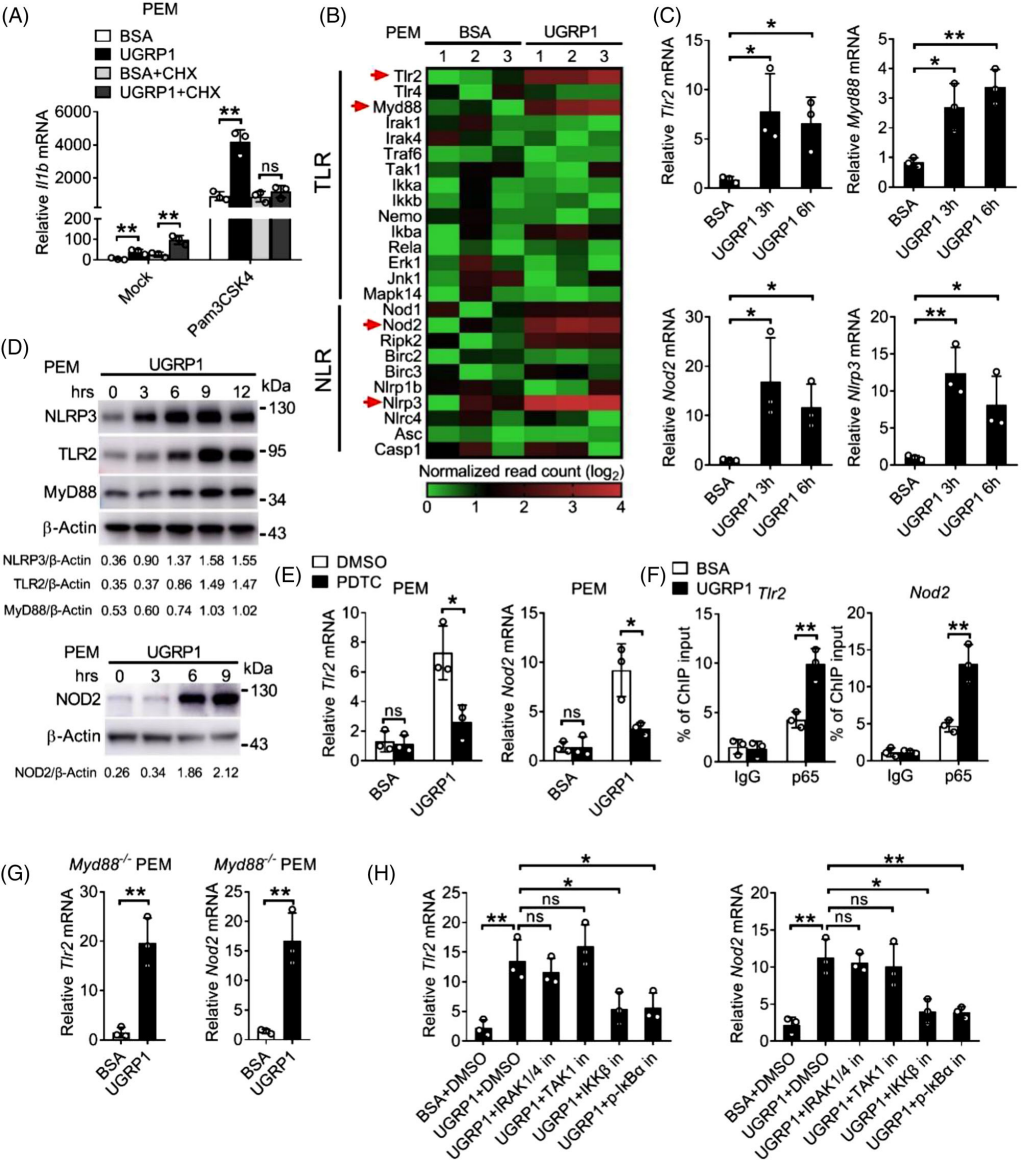
UGRP1 enhances expression of TLR2, NOD2, MyD88 and NLRP3 by activation of NF‐κB. (A) PEMs were treated with the protein synthesis inhibitor CHX (10 μM) for 1 h followed by Pam3CSK4 stimulation with BSA or UGRP1 (0.5 μg/ml) for 6 h to measure *Il1b* mRNA by qRT‐PCR (*n* = 3). (B) The relative mRNA levels of gene from TLRs and NLRs pathway in PEMs after UGRP1 (0.5 μg/ml) treatment for 3 h (*n* = 3). (C) PEMs were treated with UGRP1 (0.5 μg/ml) for 3 or 6 h to check *Tlr2, MyD88, Nod2* and *Nlrp3* mRNA by qRT‐PCR (*n* = 3). (D) PEMs were treated with UGRP1 (0.5 μg/ml) for the indicated periods to check NLRP3, TLR2, MyD88 and NOD2 protein levels by immunoblot analysis. (E) PEMs were treated with the NF‐κB inhibitor PDTC (100 μM) for 1 h followed by BSA or UGRP1 (0.5 μg/ml) treatment for 6 h to measure *Tlr2* and *Nod2* mRNA by qRT‐PCR (*n* = 3). (F) PEMs were treated with UGRP1 (0.5 μg/ml) for 1 h and the relative amount of *Tlr2* and *Nod2* DNA binding to p65 was analysed by ChIP assay. (G) MyD88 KO PEMs were treated with UGRP1 (0.5 μg/ml) for 6 h to check *Tlr2* and *Nod2* mRNA by qRT‐PCR (*n* = 3). (H) PEMs were treated with IRAK1/4 inhibitor IRAK‐1‐4 Inhibitor I (10 μM), TAK1 inhibitor Takinib (10 μM), IKKβ inhibitor LY2409881 trihydrochloride (5 μM) or p‐IκBα inhibitor BAY 11–7082 (20 μM) for 1 h followed by UGRP1 (0.5 μg/ml) treatment for 6 h to measure *Tlr2* and *Nod2* mRNA by qRT‐PCR (*n* = 3). *p < .05, **p < .01 and ***p < .001, using a two‐tailed, unpaired Student's *t*‐test (G), one‐way analysis of variance (ANOVA) with Holm–Sidak's multiple comparisons test (C and H) or two‐way ANOVA with Holm–Sidak's multiple comparisons test (A and E). Data from at least three independent experiments (mean ± SD) or representative data (D). Please also see Figure S3

In order to identify whether increased production of pro‐inflammatory cytokine, which resulted from UGRP1 together with agonist stimulation, was dependent on synthesis of new protein. Protein synthesis inhibitor cycloheximide (CHX) was used to treat PEMs with UGRP1 and Pam3CSK4 stimulation. Similar *Il1b* mRNA levels were observed in CHX and Pam3CSK4 stimulated PEMs with or without UGRP1 treatment (Figure 3A), which implied that UGRP1 enhanced expression of pro‐inflammatory cytokine was dependent on new protein synthesis. In order to identify the UGRP1‐induced protein, we used mRNA sequencing analysis as well as qRT‐PCR to check UGRP1‐treated macrophages, and increased mRNA levels of *Tlr2, Myd88, Nod2* and *Nlrp3*, but not *Tlr4*, were observed (Figure 3B,C and Figure S3A). Immunoblot analysis also confirmed enhanced expression of TLR2, MyD88, NOD2 and NLRP3 followed by UGRP1 treatment (Figure 3D), and we observed increased TLR2 surface expression and NLRP3 total expression with FACS assay (Figure S3B). In addition, UGRP1 treatment also increased expression of MyD88 in phorbol‐12‐myristate‐13‐acetate (PMA)‐induced human THP‐1 cells (Figure S3C).

In consideration of enhanced NF‐κB activation induced by UGRP1, we used PDTC to incubate with PEMs followed by UGRP1 treatment. Reduced expression of TLR2, NOD2 and MyD88 was observed after PDTC treatment (Figure 3E and Figure S3D). The occupation of the *Tlr2* and *Nod2* promoter region by the NF‐κB subunit p65 was detected in UGRP1‐treated PEMs using chromatin immunoprecipitation (ChIP) assays (Figure 3F), which indicated that UGRP1 enhanced TLR2 and NOD2 expression by activation of NF‐κB. In order to verify whether UGRP1 motivated the same signalling proteins as TLRs to activate NF‐κB, MyD88 KO PEMs were treated with UGRP1 and increased TLR2 and NOD2 expression was still observed (Figure 3G and Figure S3E), although Pam3CSK4 stimulated MyD88 KO PEMs showed almost no *Il6* expression (Figure S3F). Then, various inhibitors of TLRs‐NF‐κB signalling kinases, including IRAK1/4 inhibitor (IRAK‐1‐4 Inhibitor I), TAK1 inhibitor (Takinib), IKKβ inhibitor (LY2409881 trihydrochloride) and p‐IκBα inhibitor (BAY 11–7082), were used to incubate with UGRP1‐treated PEMs, and we observed reduced *Tlr2* and *Nod2* mRNA levels followed by inhibition of IKKβ and p‐IκBα, while almost unchanged expression of *Tlr2* and *Nod2* after blocking activation of IRAK1/4 and TAK1 (Figure 3H). In addition, reduced *Il6* mRNA levels could be observed in Pam3CSK4 stimulated PEMs following any inhibitor treatment mentioned above (Figure S3G) and these inhibitors also had no effect on PEMs viability (Figure S4M). Since Pam3CSK4 could activate NF‐κB, we demonstrated that Pam3CSK4 stimulation could enhance the expression of TLR2 which would be even more with UGRP1 treatment (Figure S3H). In accordance with PEMs data, AMs from UGRP1‐deficient mice showed depressed expression of TLR2 (Figure S3I). Together, these data suggest that UGRP1 enhances expression of TLR2, NOD2 and MyD88 by activation of IKKβ and phosphorylation of IκBα. In addition, up‐regulation of NF‐κB signalling by UGRP1 is independent of activation of IRAK1/4 and TAK1, which is different from TLRs‐induced NF‐κB activation.

### 2.4 UGRP1–PDPN signalling activates RhoA to regulate TLR2‐induced inflammation

**FIGURE 4:**
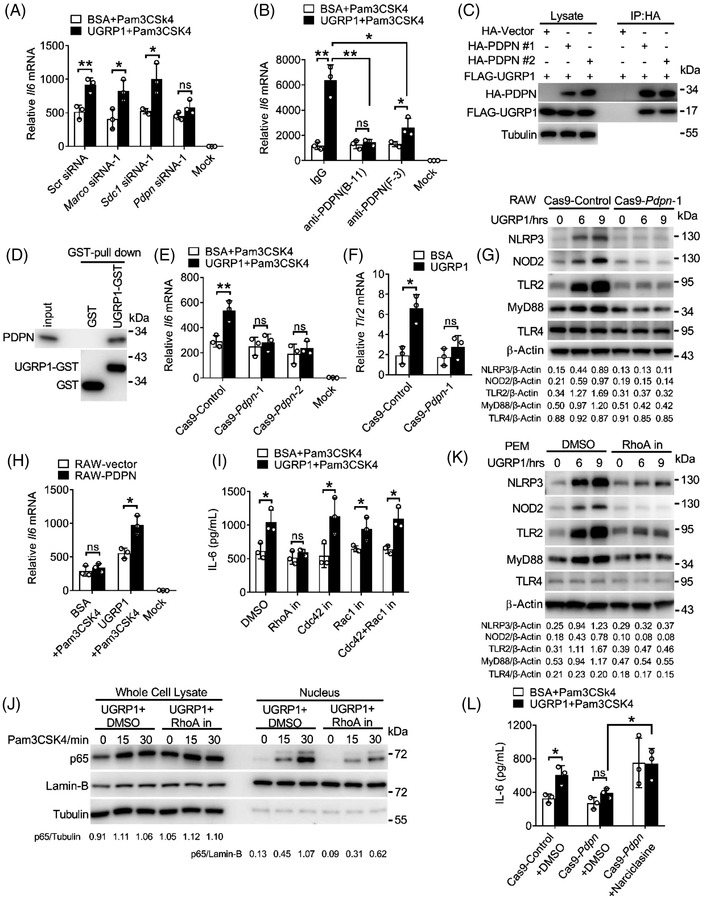
UGRP1–PDPN signalling activates RhoA to regulate TLR2‐induced inflammation. (A) PEMs were transfected with control siRNA, *Marco* siRNA, *Sdc1* siRNA or *Pdpn* siRNA followed by Pam3CSK4 (0.5 μg/ml) stimulation for 6 h with BSA or UGRP1 (0.5 μg/ml) treatment and the *Il6* mRNA levels were checked using qRT‐PCR (*n* = 3). (B) PEMs were incubated with control IgG or two PDPN antibodies (2 μg/ml) named anti‐PDPN (B‐11) or anti‐PDPN (F‐3) for 1 h followed by Pam3CSK4 (0.5 μg/ml) stimulation accompanied with BSA or UGRP1 (0.5 μg/ml) treatment for 6 h. *Il6* mRNA levels were measured by qRT‐PCR (*n* = 3). (C) HA‐Vector or two HA‐tagged PDPN clones suffixed with #1 or #2 were transfected into 293T cells respectively with FLAG‐tagged UGRP1 clone. Immunoprecipitation and immunoblot were performed with the indicated antibodies. (D) GST protein and UGRP1‐GST (2 μg) were incubated with PEMs’ lysate for 2 h followed by incubated with GST glutathione agarose for another 2 h. The samples were analysed by immunoblot. (E) Cas9‐control and Cas9‐*Pdpn*‐deficient RAW264.7 cells were stimulated by Pam3CSK4 (0.5 μg/ml) with BSA or UGRP1 (0.5 μg/ml) for 6 h to measure *Il6* mRNA levels by qRT‐PCR (*n* = 3). (F and G) Cas9‐control and Cas9‐*Pdpn*‐deficient RAW264.7 cells were treated with BSA or UGRP1 (0.5 μg/ml) for 6h to measure *Tlr2* mRNA levels by qRT‐PCR (*n* = 3; F), or for 6 or 9 h to measure NLRP3, NOD2, TLR2, MyD88 and TLR4 expression by immunoblot analysis (G). (H) RAW264.7 cells overexpressed PDPN were stimulated with Pam3CSK4 (0.5 μg/ml) for 6 h with BSA or UGRP1 (0.5 μg/ml) treatment and the *Il6* mRNA levels were checked using qRT‐PCR (*n* = 3). (I) PEMs were treated with RhoA inhibitor (CCG‐1423, 20 μM), Cdc42 inhibitor (CASIN, 20 μM), Rac1 inhibitor (1A‐116, 10 μM) or dual Cdc42/Rac1 inhibitor (MBQ‐167, 10 μM) for 1 h followed by Pam3CSK4 (0.5 μg/ml) and BSA or UGRP1 (0.5 μg/ml) treatment for 9 h to measure IL‐6 expression by ELISA (*n* = 3). (J) Immunoblot analysis of p65 in the whole cell lysate and nucleus in UGRP1 (0.5 μg/ml) and RhoA inhibitor (CCG‐1423, 20 μM) treated PEMs for 6 h followed by Pam3CSK4 (0.5 μg/ml) stimulation for the indicated periods. Tubulin were used as the whole lysate protein control, Lamin‐B served as the nucleic protein control. (K) PEMs were treated with DMSO control or RhoA inhibitor (CCG‐1423, 20 μM) for 1 h followed by UGRP1 (0.5 μg/ml) incubation for 6 or 9 h to measure NLRP3, NOD2, TLR2, MyD88 and TLR4 expression by immunoblot analysis. (L) Cas9‐control and Cas9‐*Pdpn*‐deficient RAW264.7 cells were treated with DMSO or RhoA activator (Narciclasine, 0.1 μM) for 1 h followed by UGRP1 and Pam3CSK4 treatment for 6 h to measure *Il6* mRNA levels by qRT‐PCR (*n* = 3). *p < .05, **p < .01 and ***p < .001, using two‐way analysis of variance (ANOVA) with Holm–Sidak's multiple comparisons test (A, B, E, F, H, I, L). Data from at least three independent experiments (mean ± SD) or representative data (C, D, G, J, K). Please also see Figure S4

In order to understand the underline mechanism of NF‐κB activation by UGRP1, we used mass spectrometry to search for the cell‐surface receptor which could bind UGRP1 to transfer signals into the cells. Several receptors were identified including MARCO, syndecan‐1 (SDC1) and PDPN which were also reported to combine with UGRP1.^16^, ^22^ Then, we used specific small interfering RNA (siRNA) to silence *Marco*, *Sdc1* and *Pdpn* in primary PEMs (siRNA silencing efficiency shown in Figure S4A). Similar *Il6* expression was observed in *Pdpn*‐silencing PEMs followed by Pam3CSK4 stimulation with or without UGRP1 treatment, while *Marco* or *Sdc1*‐silencing PEMs still showed increased *Il6* expression after Pam3CSK4 and UGRP1 stimulation (Figure 4A and Figure S4B). Subsequently, we used two kinds of anti‐PDPN antibodies named B‐11 and F‐3 from Santa Cruz to block PDPN and observed decreased *Il6* expression compared with control IgG treatment in PEMs after UGRP1 and Pam3CSK4 stimulation (Figure 4B). After F‐3 blocking, UGRP1 and Pam3CSK4 treatment enhanced *Il6* expression slightly compared with BSA control and Pam3CSK4 treatment, while B‐11 blocked PEMs showed similar *Il6* expression followed by Pam3CSK4 stimulation with or without UGRP1 (Figure 4B), suggesting better PDPN‐blocking effect of B‐11. On the other hand, both F‐3 and B‐11 had no effect on PEMs viability (Figure S4C). The increased expression of TLR2 after UGRP1 treatment could also be restrained by B‐11 or F‐3 blocking, with the better suppressing effect of B‐11 (Figure S4D). We then verified the interaction of FLAG‐tagged UGRP1 and HA‐tagged PDPN using immunoprecipitation in 293T cells (Figure 4C), and B‐11 could interrupt the interaction of UGRP1 and PDPN (Figure S4E). Moreover, purified GST‐tagged UGRP1 could also pull down endogenous PDPN in PEMs (Figure 4D).

Then we generated PDPN‐ablated RAW264.7 cell lines with the CRISPR (Clustered Regularly Interspaced Short Palindromic Repeats)/Cas9 system. The genetic ablation efficiency was tested (Figure S4F). UGRP1 treatment could not enhance *Il6* expression in PDPN‐deficient RAW264.7 cells after Pam3CSK4 stimulation (Figure 4E), and PDPN deficiency also suppressed enhanced translocation of p65 into nucleus (Figure S4G). Consistently, elimination of increased NLRP3, NOD2, TLR2 and MyD88 expression with constant expression of TLR4 was observed in UGRP1‐treated PDPN‐deficient RAW264.7 cells (Figure 4F,G), which showed diminished UGRP1 binding as well (Figure S4H). Then, PDPN was overexpressed in RAW264.7 cells (Figure S4I), and increased *Il6* expression was observed in PDPN‐overexpressed cells with UGRP1 and Pam3CSK4 stimulation (Figure 4H). Flow cytometry assay showed positive expression of PDPN in AMs (Figure S4J), indicating that like PEMs and RAW264.7 cells, UGRP1 could stimulate the same signalling pathway in AMs. So, we have demonstrated that UGRP1 interacts with PDPN, and this interaction could enhance the expression of TLR2, NOD2 and MyD88 to amplify NF‐κB activation.

PDPN is a small transmembrane glycoprotein, which co‐localises with members of the ERM (ezrin, radixin, moesin) protein family and triggers small GTPase RhoA activation.^23^ In order to identify whether UGRP1‐induced enhancement of IL‐6 expression was dependent on RhoA activation. RhoA inhibitor CCG‐1423 was used and we observed similar IL‐6 expression after Pam3CSK4 stimulation with UGRP1 or BSA control treatment (Figure 4I and Figure S4K). Since small GTPase proteins including Cdc42 and Rac1 were also reported to be regulated by PDPN,^24^, ^25^ we used CASIN (Cdc42 inhibitor), 1A‐116 (Rac1 inhibitor) or MBQ‐167 (dual Cdc42/Rac1 inhibitor) to inhibit Cdc42 and Rac1 activation and still observed enhanced IL‐6 expression in UGRP1‐treated PEMs compared with BSA‐treated controls after Pam3CSK4 stimulation (Figure 4I). Consistently, UGRP1‐induced enhancement of p65 translocation into nucleus was suppressed by CCG‐1423 after Pam3CSK4 stimulation (Figure 4J). Increased NLRP3, TLR2, MyD88 and NOD2 expression induced by UGRP1 alone was also restrained by CCG‐1423, with no effect on expression of TLR4 (Figure 4K and Figure S4L). In addition, these inhibitors had no effect on PEMs viability (Figure S4M). Then, RhoA activator Narciclasine was used and we observed suppressed IL‐6 expression, resulted from PDPN deficient, was recovered in Cas9‐*Pdpn* RAW264.7 cells after UGRP1 and Pam3CSK4 treatment (Figure 4L). Meanwhile, increased expression of NLRP3, NOD2, TLR2 and MyD88, resulted by RhoA activation, was eliminated by NF‐κB inhibition (Figure S4N). These results indicate that enhancement of NF‐κB activation induced by UGRP1–PDPN signalling is dependent on RhoA activation.

### 2.5 UGRP1 activates RhoA to enhance the interaction of IKKγ and IKKβ

**FIGURE 5:**
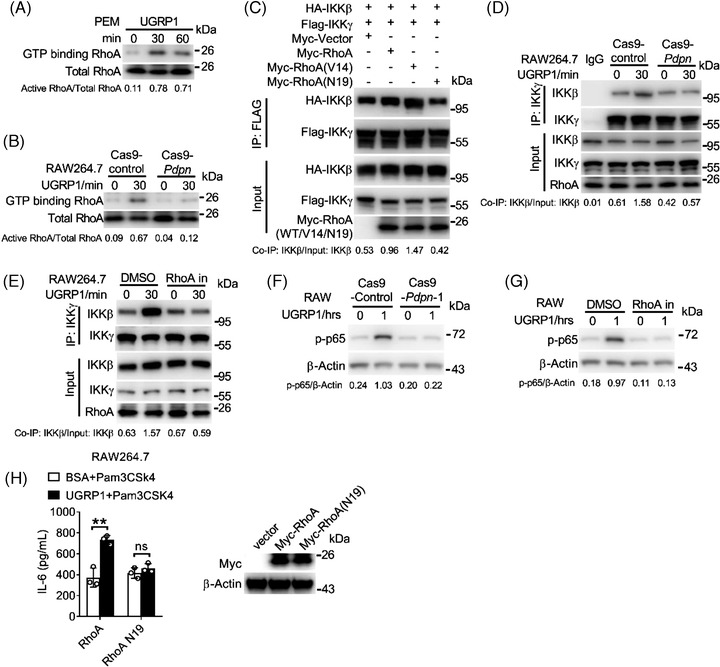
UGRP1 activates RhoA to enhance the interaction of IKKγ and IKKβ. (A and B) PEMs (A) and Cas9‐control or Cas9‐*Pdpn*‐deficient RAW264.7 cells (B) were treated with UGRP1 for indicated periods and the cell lysates were subjected to GST‐Rhotekin‐RBD pull‐down assay to precipitate the activated RhoA. (C) Myc‐RhoA, Myc‐RhoA (V14, constitutively active mutant) or Myc‐RhoA (N19, constitutively negative mutant) were transfected into 293T cells with FLAG‐IKKγ and HA‐IKKβ. Cell lysates were subjected to immunoprecipitation with anti‐FLAG. (D) Cas9‐control or Cas9‐*Pdpn*‐deficient RAW264.7 cells were treated with UGRP1 (0.5 μg/ml) for indicated periods and the cell lysates were subjected to immunoprecipitation with anti‐IKKγ. (E) RAW264.7 cells were treated with DMSO control or RhoA inhibitor (CCG‐1423, 20 μM) for 1 h followed by UGRP1 (0.5 μg/ml) stimulation for indicated periods and the cell lysates were subjected to immunoprecipitation with anti‐IKKγ. (F) Cas9‐control or Cas9‐*Pdpn*‐deficient RAW264.7 cells were treated with UGRP1 (0.5 μg/ml) for indicated periods and the cell lysates were subjected to detect phosphorylated (p‐) p65. (G) RAW264.7 cells were treated with DMSO control or RhoA inhibitor (CCG‐1423, 20 μM) for 1 h followed by UGRP1 (0.5 μg/ml) stimulation for indicated periods and the cell lysates were subjected to detect phosphorylated (p‐) p65. (H) RAW264.7 cells stably overexpressing WT RhoA or RhoA N19 mutant were stimulated by Pam3CSK4 (0.5 μg/ml) with BSA or UGRP1 (0.5 μg/ml) for 9 h. IL‐6 protein levels were measured by ELISA (*n* = 3, right pane) and overexpression of RhoA was checked by immunoblot (left panel). *p < .05, **p < .01 and ***p < .001, using two‐way analysis of variance (ANOVA) with Holm–Sidak's multiple comparisons test (H left panel). Data from at least three independent experiments (mean ± SD) or representative data (A–G, H right panel). Please also see Figure S5

We used GST‐Rhotekin‐RBD pull‐down assay to test the activation of RhoA. UGRP1‐treated PEMs showed increased RhoA activation (Figure 5A), which was also exhibited in UGRP1‐treated Cas9‐control RAW264.7 cells (Figure 5B), and PDPN deficiency restrained the enhancement of activated RhoA in UGRP1‐treated RAW264.7 cells (Figure 5B). Since the activated RhoA was reported to enhance the interaction of IKKγ and IKKβ to result in IKKβ activation,^26^ we constructed the constitutively active RhoA mutant V14 (glycine in 14 was replaced by valine) and constitutively negative RhoA mutant N19 (threonine in 19 was replaced by asparagine). Then, vector control, RhoA, V14 or N19 was transfected into 293T cells with IKKγ and IKKβ. We observed increased combination of IKKγ and IKKβ in RhoA transfected cells compared with vector control transfected cells (Figure 5C). Constitutively active mutant V14 transfected cells showed most combination of IKKγ and IKKβ, and constitutively negative mutant N19 transfected cells exhibited similar combination of IKKγ and IKKβ compared with vector transfected controls (Figure 5C). In RAW264.7 cells, enhanced combination of IKKγ and IKKβ was observed after UGRP1 treatment, and PDPN deficiency or RhoA inhibitor CCG‐1423 eliminated this increased combination (Figure 5D,E). Simultaneously, increased phosphorylation of p65 was also eliminated by PDPN deficiency or RhoA inhibitor (Figure 5F,G). Then, we also observed overexpression of constitutively negative RhoA mutant N19 abrogated enhanced expression of IL‐6 and TLR2 after UGRP1 treatment with or without Pam3CSK4 stimulation in RAW264.7 cells (Figure 5H and Figure S5A). Although PDPN was reported to interact with CLEC‐2 (C‐type‐lectin‐like‐2) to inhibit inflammation in sepsis,^27^ extra CLEC‐2 protein incubation had no effect on *Il6* and *Tnfa* expression in Pam3CSK4‐stimulated PEMs during our study (Figure S5B). Also, decreased expression of *Clec2* did not regulate *Il6* mRNA levels in Pam3CSK4‐stimulated PEMs with or without UGRP1 treatment (Figure S5C,D), and similar expression of *Tlr2* and *Nod2* was observed in scrambled and *Clec2* transfected PEMs (Figure S5E) indicating insignificant effect of CLEC‐2–PDPN signalling in our studied inflammatory pathway. Together, we have demonstrated that UGRP1 activates RhoA to enhance the combination of IKKγ and IKKβ and our data have also elucidated a model linking the UGRP1–PDPN signalling to TLR2/NOD2‐induced inflammation (Figure S5E).

### 2.6 UGRP1 deficiency protects against *S. pneumoniae*‐induced severe pneumococcal pneumonia

**FIGURE 6:**
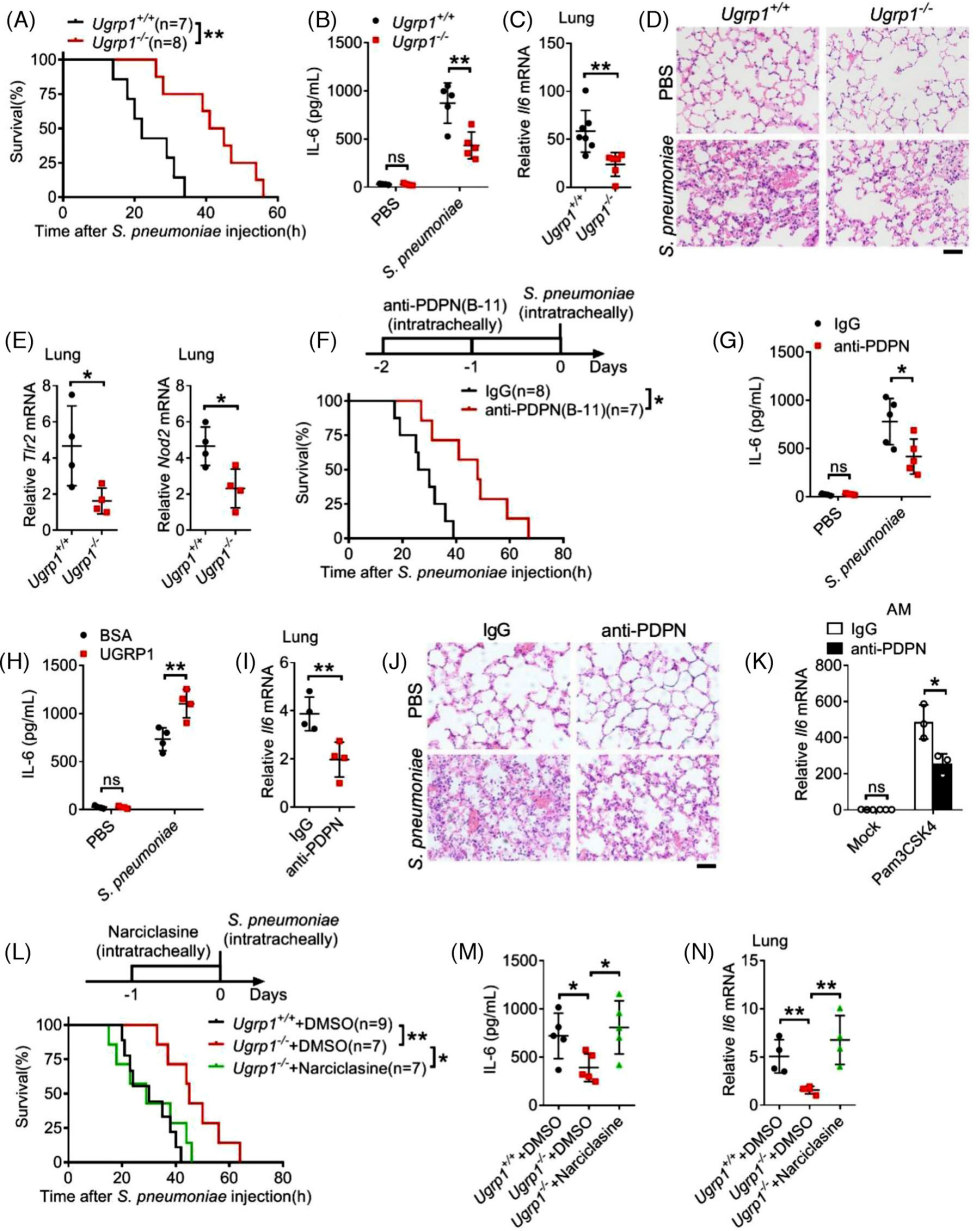
UGRP1 deficiency protects against *S. pneumoniae*‐induced pneumonia. (A) WT and UGRP1 KO mice were infected intratracheally with *S. pneumoniae* (4 × 10^5^ CFU/mouse) and the survival time was assessed. (B–E) WT and UGRP1 KO mice were infected intratracheally with *S. pneumoniae* (10^5^ CFU/mouse). IL‐6 in BAL (bronchoalveolar lavage) fluid was detected by ELISA at 1 day after inoculation (B, *n* = 5). Lungs were also harvested at 1 day post infection and *Il6* (C, *n* = 6), *Tlr2* and *Nod2* (E, *n* = 4) mRNA levels were checked by qRT‐PCR. Haematoxylin and eosin (H&E) staining of lung tissue was performed as well (D, scale bar: 20 μm). (F) WT mice pre‐treated with control IgG or anti‐PDPN (B‐11) antibody (1 μg/mouse) were infected intratracheally with *S. pneumoniae* (4 × 10^5^ CFU/mouse) to examine the survival time. (G) WT mice pre‐treated with control IgG or anti‐PDPN (B‐11) antibody (1 μg/mouse) were infected intratracheally with *S. pneumoniae* (10^5^ CFU/mouse). IL‐6 in BAL fluid was detected by ELISA at 1 day after inoculation (*n* = 5). (H) WT mice treated with BSA or UGRP1 (1 μg/mouse) for 1 day, following by infected intratracheally with *S. pneumoniae* (10^5^ CFU/mouse). IL‐6 in BAL fluid was detected by ELISA at 1 day after inoculation (*n* = 5). (I and J) WT mice pre‐treated with control IgG or anti‐PDPN (B‐11) antibody (1 μg/mouse) were infected intratracheally with *S. pneumoniae* (10^5^ CFU/mouse). Lungs were harvested at 1 day post infection and *Il6* mRNA levels were checked by qRT‐PCR (I, *n* = 4). H&E staining of lung tissue was performed as well (J, scale bar: 20 μm). (K) AMs from IgG or anti‐PDPN (B‐11) (1 μg/mouse) treated mice were stimulated with Pam3CSK4 for 6 h to check *Il6* mRNA by qRT‐PCR (*n* = 3). (L) WT and UGRP1 KO mice pre‐treated with DMSO or RhoA activator (Narciclasine, 5 μg/mouse) were infected intratracheally with *S. pneumoniae* (4 × 10^5^ CFU/mouse) to examine the survival time. (M and N) WT and UGRP1 KO mice pre‐treated with DMSO or RhoA activator (Narciclasine, 5 μg/mouse) were infected intratracheally with *S. pneumoniae* (10^5^ CFU/mouse). IL‐6 in BAL fluid was detected at 1 day after inoculation by ELISA (K, *n* = 5). Lungs were also harvested at 1 day post infection and *Il6* mRNA levels were checked by qRT‐PCR (L, *n* = 4). *p < .05, **p < .01 and ***p < .001, using a two‐tailed, unpaired Student's *t*‐test (C, E and I), one‐way analysis of variance (ANOVA) with Holm–Sidak's multiple comparisons test (M and N), two‐way ANOVA with Holm–Sidak's multiple comparisons test (B, G, H and K) or log‐rank (Mantel–Cox) test (A, F and L). Data from at least three independent experiments (mean ± SD) or representative data (D and J). Please also see Figure S6

Given that extreme NF‐κB activation plays an important role in sepsis and pneumonia, we investigated whether UGRP1 deficiency protected against severe pneumococcal pneumonia induced by *S. pneumoniae*. Extended survival time was observed in UGRP1 KO mice compared with their WT littermate controls following the intratracheal injection of *S. pneumoniae* (Figure 6A). In accord with the critical role of uncontrolled inflammatory response in pneumonia, significantly lower expression of IL‐6 in BAL fluid of UGRP1 KO mice was observed (Figure 6B). Similarly, UGRP1 KO mice exhibited less *Il6* (Figure 6C), *Tnfa* and *Il1b* (Figure S6A) mRNA levels in lungs, together with alleviative lung injuries (Figure 6D). In accordance with elevated TLR2, NOD2 and NLRP3 expression in PEMs after UGRP1 treatment, UGRP1 KO mice showed less *Tlr2*, *Nod2* (Figure 6E) and *Nlrp3* (Figure S6B) mRNA levels in lungs after *S. pneumoniae* infection. Pulmonary bacterial titres were also evaluated and UGRP1 KO mice displayed similar pulmonary bacterial loads compared with WT controls (Figure S6C), indicating that UGRP1 deficiency did not affect *S. pneumoniae* clearance. Moreover, Clodronate Liposome was used to clear resident AMs (Figure S6D), and decreased IL‐6 was still observed in BAL fluid from UGRP1 KO mice (Figure S6E), suggesting that UGRP1 could also promote other cells inflammation in lung except for AMs. Meantime, we also observed that PDPN and F4/80 (a specific marker of mature macrophages) co‐localised on the same cell in *S. pneumoniae* infected lung (Figure S6F). Then, flow cytometry was used to analyse the number of AMs and infiltrated monocytes in lung. As previous study reported,^28^ we used the markers CD11c^high^ Siglec F^high^ CD11b^low^ to discriminate AMs, and we could see similar number of AMs between WT and UGRP1 KO mice without infection (Figure S6G,H). After *S. pneumoniae* infection, similar number of infiltrated monocytes (Ly‐6C^high^ CD11b^high^) and macrophages (Ly‐6C^high^ CD11b^high^ F4/80^high^) between WT and UGRP1 KO mice was also observed (Figure S6I,J).

Next, we used anti‐PDPN (B‐11) antibody to interrupt interaction of UGRP1 and PDPN in WT mice, and extended survival time (Figure 6F) together with reduced IL‐6 concentrations in BAL fluid (Figure 6G) was observed after *S. pneumoniae* infection. In addition, extra UGRP1 protein supplement in WT mice enhances IL‐6 concentrations in BAL fluid after *S. pneumoniae* infection (Figure 6H). Anti‐PDPN (B‐11) antibody‐injected mice also showed decreased mRNA levels of *Il6* (Figure 6I), *Tnfa* and *Il1b* (Figure S6K) in lungs, accompanied by alleviative lung injuries (Figure 6J) compared with control IgG‐injected mice. In addition, anti‐PDPN (B‐11) antibody did not affect *S. pneumoniae* clearance (Figure S6L) and number of AMs (CD11c^high^ Siglec F^high^ CD11b^low^), infiltrated monocytes (Ly‐6C^high^ CD11b^high^) and macrophages (Ly‐6C^high^ CD11b^high^ F4/80^high^) (Figure S6M). AMs from B‐11 antibody‐treated mice showed decreased TLR2 expression (Figure S6N) and reduced *Il6* mRNA levels after Pam3CSK4 stimulation (Figure 6K).

Then, RhoA activator Narciclasine was intratracheally injected into UGRP1 KO mice with *S. pneumoniae* infection and shortened survival time (Figure 6L) together with enhanced IL‐6 concentrations in BAL fluid (Figure 6M) was observed compared with DMSO‐injected UGRP1 KO controls. mRNA levels of *Il6* (Figure 6N), *Tnfa* and *Il1b* (Figure S6O) also enhanced in lungs of Narciclasine‐injected UGRP1 KO mice compared with DMSO‐injected UGRP1 KO mice. Finally, we confirmed that extended survival time and reduced lung inflammation, which were resulted from UGRP1 deficiency, could be recovered by RhoA activation. These results suggest that UGRP1–PDPN signalling enhance *S. pneumoniae*‐induced inflammation by activation of RhoA.

## 3 DISCUSSION

In this study, we have showed that UGRP1 could enhance TLR2/4 and NOD2‐induced signalling and pro‐inflammatory cytokine production in macrophages. Additional UGRP1 treatment enhanced IL‐6, TNFα and IL‐1β production in macrophages with Pam3CSK4/LPS/MDP/LTA stimulation or *S. pneumoniae* infection. In vivo, UGRP1 deficiency protected mice from *S. pneumoniae*‐induced pneumonia, indicating that targeting UGRP1 could be used to protect the host from severe pneumonia.

As reported before, non‐ciliated airway epithelial (club) cells of the trachea, bronchus and bronchioles showed abundant and specific expression of UGRP1.^29^ We also detected UGRP1 expression in different organs and macrophages, and UGRP1 was only detectable in lung. *S. pneumoniae*‐infected AMs from UGRP1 KO mice showed decreased pro‐inflammatory cytokine levels compared with those from WT mice. However, UGRP1 deficiency did not affect pro‐inflammatory cytokine production in PEM and BMDM after *S. pneumoniae* infection, which indicated that the pro‐inflammatory effect of UGRP1 might be restricted only to lung. The limited effect of UGRP1 was in accord with its distribution, which showed average 50–100 times higher in BAL fluid than in serum.^21^ In consideration of dilution of UGRP1 in BAL fluid preparation, macrophage might be confronted with more than 50–100 folds concentration of UGRP1 in lung than in blood. So, our study provides an interesting example on lung specific secretory protein regulates bacterial infection and inflammation locally, which will also trigger more interest in understanding the function of other lung secretory proteins in inflammation.

In our study, we also noticed that UGRP1‐treated macrophages showed increased expression of NLRP3, which could take part in the formation of inflammasome and activate caspase‐1 to catalyse maturation of IL‐1β and IL‐18.^30^, ^31^ NLRP3 inflammasome could recognise RNA particles from the influenza virus and result in an inflammatory response which lead to the recruitment of immune cells, such as monocytes and neutrophils.^32^, ^33^ The levels of lung injury were decreased in mechanically ventilated NLRP3 deficiency mice.^34^ It was also reported that NLRP3 inflammasome activity was closely associated with tumor progression in lung cancer.^35^ In consideration of multiple function of NLRP3 inflammasome, the role of UGRP1 should also be investigated extensively in lung inflammation, injury and cancer.

UGRP1 was verified to be a pro‐inflammatory protein in our study, and mainly played the pro‐inflammatory role in lung, where AMs are regulated routinely by UGRP1. UGRP1 deficiency results in the decreased TLR2 expression in AMs and reduced pro‐inflammatory cytokines production after *S. pneumoniae* infection. Although UGRP1 promotes the inflammation in AMs, a considerable influence could negatively regulate AMs in the microenvironment of the airway. For example, SPA, which is abundant in the surface fluid of the epithelium, promotes an anti‐inflammatory effect in the lungs by inhibiting the binding of TLR ligands to TLR2 and TLR4.^36^ Because of the positive and the negative regulation in lung, AMs are essential for steady state but are also ready to initiate an intense inflammatory response to some pathogen.

Since 2020, the coronavirus infectious disease 2019 (COVID‐19), which is caused by a severe acute respiratory syndrome coronavirus 2 (SARS‐CoV‐2), has spread at the global scale.^37^ Higher levels of IL‐6 and TNFα were observed in deceased patients infected with COVID‐19 compared with patients who had restored from the disease,^38^ and an uncontrolled cytokine response has also been observed in COVID‐19 patients.^39^ As the RNA virus, SARS‐CoV‐2 could be recognised by RIG‐I and activate IRF3 and NF‐κB pathways, which promote the production of IFNβ, IL‐6, TNFα and so on.^40^, ^41^ So UGRP1, as a pro‐inflammatory protein in lung, will attract more attention on its role in regulation of cytokine storm induced by COVID‐19.

PDPN is a cell‐surface mucin‐type transmembrane glycoprotein.^42^ Previous studies have indicated that PDPN played a role in regulation of a variety of cellular signalling pathways, including proliferation, contractility, migration, chronic inflammation and cancer, which was dependent on binding of PDPN to its ligands.^43^ Binding of soluble CLEC‐2 to PDPN restrains the inflammation induced by Th17.^44^ The interaction of PDPN with CCL21 in fibroblast‐like reticular cells is relevant with the development of specialised T cells.^45^ In this work, we demonstrated that binding of UGRP1 and PDPN‐activated RhoA to enhance the interaction of IKKγ and IKKβ in macrophages. The increased interaction of IKKγ and IKKβ activated NF‐κB to promotes expression of TLR2, MyD88, NOD2 and NLRP3, which augmented the inflammation induced by *S. pneumoniae* infection. So, we identified a new ligand which bond PDPN to regulate the inflammation in macrophage.

In summary, we have provided convinced evidence that the specific lung expressed secretion protein UGRP1 binds macrophage cell surface receptor PDPN to modulate a novel inflammation pathway that enhances NF‐κB phosphorylation in an TLR2/4‐independent manner in response to *S. pneumoniae* infection. Because of the extracellular location of UGRP1 and the cell surface location of PDPN, specific antibodies could bind UGRP1 or PDPN directly to regulate this signalling pathway. Because of the important role of UGRP1, PDPN and the pro‐inflammatory cytokines including IL‐6 and TNFα in inflammation, alternative site for drug design could be provided by this study.

## 4 METHODS

Following are the reagents or resources used in this study along with their respective sources.

**Table:** 

Reagent and resource	Source	Identifier
Antibodies		
Rabbit anti‐pp65 (Ser536)	Cell Signaling Technology	Cat# 3033
Rabbit anti‐p65	Cell Signaling Technology	Cat# 8242
Rabbit anti‐IκBα	Cell Signaling Technology	Cat# 4812
Rabbit anti‐pIKKα/β (Ser176/180)	Cell Signaling Technology	Cat# 2697
Rabbit anti‐α‐Tubulin	Cell Signaling Technology	Cat# 2125
Rabbit anti‐IKKβ	Cell Signaling Technology	Cat# 8943
Rabbit anti‐RhoA	Cell Signaling Technology	Cat# 2117
Mouse anti‐Myc	Cell Signaling Technology	Cat# 2276
Rabbit anti‐Lamin B1	Abcam	Cat# ab229025
Mouse anti‐β‐Actin‐HRP	Abcam	Cat# ab20272
Rabbit anti‐UGRP1	Abcam	Cat# ab181853
Rabbit anti‐MyD88	Abcam	Cat# ab219413
Rabbit anti‐TLR2	Abcam	Cat# ab209217
Rabbit anti‐NLRP3	Abcam	Cat# ab263899
Rabbit anti‐IKKγ	Abcam	Cat# ab178872
Rat anti‐CD11b‐APC	Invitrogen	Cat# 17‐0112‐82
Rat anti‐F4/80‐FITC	Invitrogen	Cat# 11‐4801‐82
Rat anti‐CD4‐FITC	Invitrogen	Cat# 11‐0041‐82
Rat anti‐CD8‐APC	Invitrogen	Cat# 17‐0081‐82
Rat anti‐B220‐PE	Invitrogen	Cat# 12‐0452‐82
Rat anti‐NOD2	Invitrogen	Cat# 14‐5858‐82
Rat anti‐Ly‐6C‐ PerCP‐Cyanine5.5	Invitrogen	Cat# 45‐5932‐80
Armenian hamster anti‐CD11c‐APC	Invitrogen	Cat# 17‐0114‐82
Rat anti‐Siglec F‐Alexa Fluor 488	Invitrogen	Cat# 53‐1702‐82
Mouse anti‐HA	Sigma–Aldrich	Cat# H3663
Mouse anti‐FLAG	Sigma–Aldrich	Cat# F3165
Mouse anti‐PDPN (F‐3)	Santa Cruz	Cat# sc‐376962
Mouse anti‐PDPN (B‐11)	Santa Cruz	Cat# sc‐166906
Mouse anti‐GST	YEASEN	Cat# 30901ES10
Bacterial		
*Streptococcus pneumoniae*	ATCC	Cat# 6303
Chemicals, peptides and recombinant proteins		
Narciclasine	MCE	Cat# HY‐16563
CCG‐1423	MCE	Cat# HY‐13991
CASIN	MCE	Cat# HY‐12874
1A‐116	MCE	Cat# HY‐104064
MBQ‐167	MCE	Cat# HY‐112842
IRAK‐1‐4 Inhibitor I	MCE	Cat# HY‐13329
Takinib	MCE	Cat# HY‐103490
LY2409881 trihydrochloride	MCE	Cat# HY‐B0788A
BAY 11–7082	MCE	Cat# HY‐13453
PDTC	MCE	Cat# HY‐18738
Pam3CSK4	InvivoGen	Cat# tlrl‐pms
LTA	InvivoGen	Cat# tlrl‐slta
MDP	InvivoGen	Cat# tlrl‐mdp
LPS	Sigma–Aldrich	Cat# L3012
GST	Abcam	Cat# ab81793
CLEC‐2	Sino Biological	Cat# 50306‐M01H
GST‐UGRP1	Abcam	Cat# ab239571
CHX	Millipore	Cat# 5.08739
UGRP1	Novoprotein	Cat# C190
Mouse M‐CSF	PeproTech	Cat# 315‐02
3X FLAG Peptide	YEASEN	Cat# 20571ES11
Protein G Agarose Resin	YEASEN	Cat# 36406ES08
Anti‐FLAG Affinity Gel	YEASEN	Cat# 20585ES01
GSTSep Glutathione Agarose Resin	YEASEN	Cat# 20508ES10
Puromycin	YEASEN	Cat# 60210ES25
Chemiluminescent HRP Substrate	Millipore	Cat# WBKLS0500
Lipofectamine RNAiMAX	Invitrogen	Cat# 13778030
Lipofectamine 2000	Invitrogen	Cat# 11668030
TRIzol	Invitrogen	Cat# 15596018
PrimeScript RT reagent Kit	Takara	Cat# RR047A
TB Green Premix Ex Taq	Takara	Cat# RR420Q
Critical Commercial Assays		
IL‐6 Mouse ELISA Kit	Invitrogen	Cat# 88‐7064‐22
TNFα Mouse ELISA Kit	Invitrogen	Cat# 88‐7324‐22
Cell Counting Kit (CCK‐8)	YEASEN	Cat# 40203ES60
Active Rho Detection Kit	Cell Signaling Technology	Cat# 8820S
Nucleoprotein Extraction Kit	Sangon Biotech	Cat# C500009
Cell lines		
HEK293T cells	ATCC	Cat# ACS‐4500
THP‐1	ATCC	Cat# TIB‐202
RAW264.7 macrophages	Laboratory of Hongyan Wang	N/A
Organisms/strains		
Mouse: C57BL/6J	Shanghai Model Organisms	N/A
Mouse: B6 *Ugrp1^−/−^*	This paper	N/A
Mouse: B6 *Myd88^−/−^*	GemPharmatech	N/A
Software and algorithms		
GraphPad Prism version 8.0	GraphPad Software	https://www.graphpad.com/
FlowJo v10	FlowJo	https://www.flowjo.com/solutions/flowjo
QuantStudio Real‐Time PCR Software	ABI	N/A

**Table:** 

Recombinant DNA	Cloning primers	Cloning sites	Accession number
pcDNA3.1‐FLAG‐UGRP1	F: 5′‐GGAATTCGCCACCATGAAGCTGGTAACTATCTTC‐3′	*Xho*I	NM_054023.5
	R: 5′‐TTGCGGCCGCTCACTTGTCGTCATCGTCTTTGTAGTCCACCAAGTGTGATAGCGCCT‐3′	*Not*I	
pcDNA3.1‐HA‐PDPN	F: 5′‐GGAATTCGCCACCATGTGGACCGTGCCAGTGT‐3′	*Eco*RI	NM_010329.3
	R: 5′‐GCTCTAGATTAAGCGTAATCTGGAACATCGTATGGGTACTCGAGGGGCGAGAACCTTCCAGAAATC‐3′	*Xba*I	
pcDNA3.1‐FLAG‐IKKγ	F: 5′‐ACCGCTCGAGCCACCATGTATATCAGGTACTGCTGTGA‐3′	*Xho*I	NM_001136067.2
	R: 5′‐GCATGCGGCCGCTCTCTATGCACTCCATGACATG‐3′	*Not*I	
pcDNA3.1‐HA‐IKKβ	F: 5′‐ACCGCTCGAGCCACCATGAGCTGGTCACCGTCCCTC‐3′	*Xho*I	NM_001159774.1
	R: 5′‐GCATGCGGCCGCTGTCACAGGCCTGCTCCAGGC‐3′	*Not*I	
pcDNA3.1‐Myc‐RhoA	F: 5′‐ACCGCTCGAGCCACCATGGCTGCCATCAGGAAGAAAC‐3′	*Xho*I	NM_001313961.1
	R: 5′‐GCATGCGGCCGCGCCAAGATGAGGCACCCAGACT‐3′	*Not*I	
pcDNA3.1‐Myc‐RhoA V14	F: 5′‐GTAGCTTGTGGTAAGACATGC‐3′	*Xho*I	NM_001313961.1
	R: 5′‐ATCACCAACAATCACCAGTTTC‐3′	*Not*I	
pcDNA3.1‐Myc‐RhoA N19	F: 5′‐AATTGCTTGCTCATAGTCTTCA‐3′	*Xho*I	NM_001313961.1
	R: 5′‐CTTACCACAAGCTCCATCAC‐3′	*Not*I	
pLVX‐Myc‐RhoA	F: 5′‐ACCGCTCGAGCCACCATGGCTGCCATCAGGAAGAAAC‐3′	*Xho*I	NM_001313961.1
	R: 5′‐GCATGCGGCCGCGCCAAGATGAGGCACCCAGACT‐3′	*Not*I	
pLVX‐Myc‐RhoA N19	F: 5′‐ACCGCTCGAGCCACCATGGCTGCCATCAGGAAGAAAC‐3′	*Xho*I	NM_001313961.1
	R: 5′‐GCATGCGGCCGCGCCAAGATGAGGCACCCAGACT‐3′	*Not*I	

### 4.1 Mice

3.6 kbp genomic sequence including the whole UGRP1 gene was replaced by 2.3 kbp DNA fragment containing Neomycin cassette to generated UGRP1 KO mice, using a recombineering‐based method (Shanghai Model Organism). Genotyping was performed with the following primers: forward, 5′‐AGCATTCCTCTCAAACCCAACAG‐3′ and reverse, 5′‐GCATTCCTTGGTTTTTGTGTCA‐3′. The mice were on a C57BL/6 background. MyD88‐KO(B6/JGpt‐Myd88^em1Cd^/Gpt) mice were purchased from Gempharmatech (Nanjing, China). All mice were raised under specific‐pathogen‐free conditions with approval of the National Institute for Viral Disease Control and Prevention. Mice were randomly distributed to different experimental groups with the same age (8–12 weeks), weight and sex. We determined the number of used mice with statistical methods and advice from related publications. All animal experiments were in agreement with the animal research related ethical regulations under the approvement of the committee for humane treatment of animals at Shanghai Jiao Tong University School of Medicine.

### 4.2 Bacteria

*S. pneumoniae* used in this study was got from ATCC (#6303) and stored at −80°C in Todd‐Hewitt Broth (THB, BD Biosciences) with 10% glycerol. Freshly grown colonies were obtained from 5% sheep blood agar plates (Yingxinbio) with overnight incubation and subsequently suspended in THB for 37°C incubation. Two hours later, bacteria got to logarithmic growth and were pelleted and resuspended in phosphate‐buffered saline (PBS). Serial dilutions were then used to assessed the bacterial concentrations.

### 4.3 *S. pneumoniae* infection and anti‐PDPN (B‐11) antibody or RhoA activator (Narciclasine) treatment

Under anaesthesia (chloral hydrate), WT or UGRP1 KO mice were intratracheally injected with 10^5^ or 4 × 10^5^ colony forming unit (CFU) of *S. pneumoniae* or PBS. Mice infected with 4 × 10^5^ CFU were observed for survival data. Mice infected with 10^5^ CFU were sacrificed at 1 day after infection. BAL fluid was collected and incubated on 5% sheep blood agar plates to determine the number of bacteria. BAL fluid was also used for IL‐6 measurement and lungs were collected for H&E staining. Anti‐PDPN (B11) antibody (1 μg/mouse) or RhoA activator (Narciclasine, 5 μg/mouse) were injected intratracheally into mice at the appointed time, followed by intratracheally inoculation of 10^5^ or 4 × 10^5^ CFU of *S. pneumoniae*. Then, mice were monitored for survival time.

### 4.4 Cell culture

Cells were cultured at 37°C under 5% CO_2_. PEMs were gathered from mice injected (intraperitoneal injection) with 3 ml 3% Brewer thioglycollate medium as previously described.^7^ RAW264.7 cells were gifts from H. Wang (SIBCB, CAS). PEMs, 293T and RAW264.7 cells were cultured in complete DMEM added with 10% (v/v) FBS, l‐glutamine (2 mM), and penicillin–streptomycin (100 U/ml). THP‐1 cells were cultured in complete RPMI 1640 medium and PMA 300 ng/ml, 12 h) was used to induce THP‐1 to differentiate into macrophages. BMDMs were generated from bone marrow cells incubated with 30% L929‐conditioned media for a week.

### 4.5 siRNA silence and qRT‐PCR

Lipofectamine™ RNAiMAX Transfection Reagent (Invitrogen) was used to transfected siRNAs (Gene Pharma) into PEMs. Total RNA was extracted from cells with TRIzol (Invitrogen) and cDNA was generated with PrimeScript™ RT reagent Kit with gDNA Eraser (Takara). qRT‐PCR was performed on a QuantStudio™ 6 Flex Real‐Time PCR System (ABI) with TB Green^®^ Premix Ex Taq™ (Takara). The relative mRNA levels, normalised to the 18s RNA, were calculated by the ∆∆Ct method. Sequences of all siRNA are listed in Table 1; all qRT‐PCR primers are listed in Table 2, and primers for qRT‐PCR used in ChIP assays are listed in Table 3.

**TABLE 1:** siRNA sequences used in this study

Gene	Sequence
Mouse *Marco* siRNA‐1	Sense: 5′‐GGGUCAAAAAGGCGAAUCU‐3′
	Antisense: 5′‐AGAUUCGCCUUUUUGACCC‐3′
Mouse *Marco* siRNA‐2	Sense: 5′‐CCUCACUCAAAAUCCAGAG‐3′
	Antisense: 5′‐CUCUGGAUUUUGAGUGAGG‐3′
Mouse *Sdc1* siRNA‐1	Sense: 5′‐GAACAAGACUUCACCUUUG‐3′
	Antisense: 5′‐CAAAGGUGAAGUCUUGUUC‐3′
Mouse *Sdc1* siRNA‐2	Sense: 5′‐GCAAAUUGUGGCUGUAAAU‐3′
	Antisense: 5′‐AUUUACAGCCACAAUUUGC‐3′
Mouse *Pdpn* siRNA‐1	Sense: 5′‐GUUCUCCCAACACAUCUGA‐3′
	Antisense: 5′‐UCAGAUGUGUUGGGAGAAC‐3′
Mouse *Pdpn* siRNA‐2	Sense: 5′‐GCUGCAUCUUUCUGGAUAA‐3′
	Antisense: 5′‐UUAUCCAGAAAGAUGCAGC‐3′
Mouse *Tlr2* siRNA‐1	Sense: 5′‐GAUAAUCACCUAUCUAGUU‐3′
	Antisense: 5′‐ AACUAGAUAGGUGAUUAUC‐3′
Mouse *Tlr2* siRNA‐2	Sense: 5′‐CACUAUCCGGAGGUUGCAU‐3′
	Antisense: 5′‐AUGCAACCUCCGGAUAGUG‐3′
Mouse *Tlr4* siRNA‐1	Sense: 5′‐GAUGAAAGGAAUCUGGAAA‐3′
	Antisense: 5′‐UUUCCAGAUUCCUUUCAUC‐3′
Mouse *Tlr4* siRNA‐2	Sense: 5′‐GGACUAUGUGAUGUGACCA‐3′
	Antisense: 5′‐UGGUCACAUCACAUAGUCC‐3′
Mouse *Nod2* siRNA‐1	Sense: 5′‐GCACAUUACCUUCCAGUGC‐3′
	Antisense: 5′‐GCACUGGAAGGUAAUGUGC‐3′
Mouse *Nod2* siRNA‐2	Sense: 5′‐GGGCACCUGAAGUUGACAU‐3′
	Antisense: 5′‐AUGUCAACUUCAGGUGCCC‐3′
Mouse *Clec2* siRNA‐1	Sense: 5′‐GCUUUAGUUCUGCUGAUCU‐3′
	Antisense: 5′‐AGAUCAGCAGAACUAAAGC‐3′
Mouse *Clec2* siRNA‐2	Sense: 5′‐CAAGAAAUUCUGCCAAGAG‐3′
	Antisense: 5′‐CUCUUGGCAGAAUUUCUUG‐3′

**TABLE 2:** Primers for qRT‐PCR used in this study

Gene	Sequence
Mouse	
*Ugrp1*	F: 5′‐ACTGCCCTTCTCATCAACCG‐3′
	R: 5′‐CAGTCCTGTCACCAGATGTTC‐3′
*Marco*	F: 5′‐ACAGAGCCGATTTTGACCAAG‐3′
	R: 5′‐CAGCAGTGCAGTACCTGCC‐3′
*Tnfα*	F: 5′‐AGTGACAAGCCTGTAGCCC‐3′
	R: 5′‐GAGGTTGACTTTCTCCTGGTAT‐3′
*Il6*	F: 5′‐TGTATGAACAACGATGATGCACTT‐3′
	R: 5′‐ACTCTGGCTTTGTCTTTCTTGTTATCT‐3′
*Il1b*	F: 5′‐GCAACTGTTCCTGAACTCAACT‐3′
	R: 5′‐ATCTTTTGGGGTCCGTCAACT‐3′
*Il10*	F: 5′‐CAGGGATCTTAGCTAACGGAAA‐3′
	R: 5′‐GCTCAGTGAATAAATAGAATGGGAAC‐3′
*Tlr4*	F: 5′‐ATGGCATGGCTTACACCACC‐3′
	R: 5′‐GAGGCCAATTTTGTCTCCACA‐3′
*Tlr2*	F: 5′‐GCAAACGCTGTTCTGCTCAG‐3′
	R: 5′‐AGGCGTCTCCCTCTATTGTATT‐3′
*Nod2*	F: 5′‐CAGGTCTCCGAGAGGGTACTG‐3′
	R: 5′‐GCTACGGATGAGCCAAATGAAG‐3′
*Nlrp3*	F: 5′‐ATTACCCGCCCGAGAAAGG‐3′
	R: 5′‐TCGCAGCAAAGATCCACACAG‐3′
*Sdc1*	F: 5′‐CTTTGTCACGGCAGACACCTT‐3′
	R: 5′‐GACAGAGGTAAAAGCAGTCTCG‐3′
*Pdpn*	F: 5′‐ACCGTGCCAGTGTTGTTCTG‐3′
	R: 5′‐AGCACCTGTGGTTGTTATTTTGT‐3′
*Myd88*	F: 5′‐TCATGTTCTCCATACCCTTGGT‐3′
	R: 5′‐AAACTGCGAGTGGGGTCAG‐3′
*Clec2*	F: 5′‐AACATCAAGCCCCGGAAACAA‐3′
	R: 5′‐GCCACGAGTCCAACAACCA‐3′
18s RNA	F: 5′‐AGTTCCAGCACATTTTGCGAG‐3′
	R: 5′‐TCATCCTCCGTGAGTTCTCCA‐3′

**TABLE 3:** Primers for qRT‐PCR used in ChIP assays

*Nod2* site1	F: 5′‐CACGGTGTTGACAGGCTAGT‐3′
	R: 5′‐TATTGTGCTGGCCTCTGCTC‐3′
*Nod2* site2	F: 5′‐GGGAACTAAACAACGCTGGC‐3′
	R: 5′‐CTGGGTGGTGTTAGGGCATT‐3′
*Tlr2* site1	F: 5′‐GGTCAAGGTGTGCTATCCCC‐3′
	R: 5′‐GTGCCAGTCACAGGGAGTTT‐3′
*Tlr2* site2	F: 5′‐CTCTGCATCCAATCTCGCCT‐3′
	R: 5′‐CCATAGCTGCATCGCAGAGT‐3′
Negative control	F: 5′‐CATCCAGGAGCCACTGAAAT‐3′
	R: 5′‐GACATGGACGCTACCTGCTC‐3′

### 4.6 Cas9‐sgRNA was used to generate PDPN‐deficient RAW264.7 cells

PDPN‐deficient RAW264.7 cells were produced by CRISPR‐associated protein‐9 nuclease (Cas9)‐mediated genome engineering as previously described.^46^ Briefly, PDPN‐sgRNA was ligated to lentiCRISPR v2 plasmid (Addgene) with puromycin resistance. Lentivirus was harvested in the medium of 293T cells after transfected with lentiCRISPR‐*Pdpn*‐sgRNA, psPAX2 (addgene) and pMD2.G (addgene) for 36 h. RAW264.7 cells were infected with the lentivirus for 36 h, followed by cultured in complete DMEM with puromycin (5 μg/ml; YEASEN) for 48 h. The surviving cells were seeded into 96‐well plates as single colony. Genome DNA from single colony was extracted and Sanger sequencing was used to analyse the single colony genotype. The PDPN‐sgRNA sequences are shown below:

PDPN sgRNA set1, 5′‐AACCCAGAACAACACTGGCA‐3′;
PDPN sgRNA set2, 5′‐GACCGTGCCAGTGTTGTTCT‐3′

### 4.7 AMs isolation

AMs were isolated as previously reported. Briefly, the mouse was euthanised; then, the skin and muscles were removed to expose the trachea. A small incision was made below the larynx, and this incision was inserted by a blunted 20‐G cannula (201483; Braun, Mersongen, Germany) toward the lungs. Then, 0.5 ml PBS with 2% FBS were admitted into the lungs by syringe. The lungs were washed three times, and BAL fluid was collected. RBCs were removed by RBC lysis buffer (NH_4_Cl), and the remaining cells were filtered through a 70 μm cell strainer. After centrifugation, AMs were collected.

### 4.8 Immunoprecipitation and immunoblot

Using Lipofectamine™ 2000 Transfection Reagent, 10 μg of control‐vector plasmid or the suitable expression plasmids was transfected into 293T cells (5 × 10^6^) cultured in 10 cm dishes. Thirty‐six hours later after transfection, 1% NP‐40 lysis buffer (20 mM Tris–HCl, 1% (v/v) NP‐40, 137 mM NaCl, 10% (v/v) glycerol and 2 mM EDTA) was used to lyse 293T cells together with Protease Inhibitor Cocktail (Sigma–Aldrich), NaF and Na_3_VO_4_ followed by centrifugation. Anti‐FLAG or indicated antibodies with Protein G Agarose Resin (YEASEN) were incubated with the collected supernates. Cold 1% NP‐40 lysis buffer was used to wash the resins for three times. Then, the resins were suspended with 2 × loading buffer (4% SDS, 10% (v/v) 2‐mercaptoehtanol, 20% (v/v) glycerol, 0.004% bromophenol blue, 125 mM Tris–HCl) and boiled for 10 min. Subsequently, sodium dodecyl sulphate polyacrylamide gel electrophoresis (SDS‐PAGE) was operated to separate the samples and the samples were transferred to a polyvinylidene fluoride membrane which was then blocked by 5% BSA in tris‐buffered saline and Tween 20 (TBST) at room temperature for 1 h. After incubated with primary antibodies and washed three times with TBST, the membrane was placed with corresponding horseradish peroxidase‐labelled secondary antibodies and detected with Chemiluminescent HRP substrate (Millipore). Amersham Imager 600 (GE) was used to record the digital images. Endogenous co‐immunoprecipitation experiments were operated with lysate of 1 × 10^7^ cells in 1 ml lysis buffer and the blot experiments were subsequently performed as described above.

### 4.9 Mass spectrometry analysis

To identify the receptor of UGRP1, purified FLAG‐UGRP1 protein (5 μg) was incubated with the lysate of PEMs for 2 h at 4°C in the lysis buffer (20 mM Tris–HCl, 1% (v/v) NP‐40, 137 mM NaCl, 10% (v/v) glycerol, 2 mM EDTA and protease inhibitor cocktail, pH 7.5), followed by incubated with the Anti‐FLAG Affinity Gel (YEASEN) for 2 h at 4°C. After three washes and elution with the FLAG peptides, the proteins were used for the mass spectrometry analysis.

### 4.10 mRNA sequencing

PEMs were treated with BSA and UGRP1 respectively and RNA extract Kit (Qiagen, Germany) was used to extract total RNA according to the manufacturer's instruction. Sequencing libraries were established with 1 μg total RNA per sample, from which poly‐T oligoattached magnetic beads were used to purify mRNA. With an Illumina HiSeq platform, we sequenced the library preparations and made 150 bp paired‐end reads. Homo sapiens sequence (http://ftp://ftp.ensembl.org/pub/release‐98/fasta/homo_sapiens/) with HISAT2 v2.0.5. was used to map the paired‐end filtered reads. Each gene was calculated fragments per kilobase of transcript per million mapped fragments to normalise the read count. Finally, the different expression of the two groups was analysed with the DESeq R package (1.16.1).

### 4.11 Cell Counting Kit‐8 assay

Cell Counting Kit‐8 (CCK‐8) assay was operated with the manufacturer's instruction. Briefly, cells were seeded in a 96‐well plate and incubated with indicated inhibitors and antibodies. Then, we added the same volume of CCK‐8 solution to the plate which was subsequently incubated at 37°C (5% CO_2_) for 1 h. Thermo Scientific Multiskan Spectrum machine was used to measure the optical density at 450 nm.

### 4.12 GST pull down

UGRP1‐GST (2 μg; Abcam) or GST protein (2 μg; YEASAN) were incubated with PEMs’ lysate at 4°C for 2 h in 200 μl pull‐down buffer (20 mM Tris–HCl, 100 mM NaCl, 5 mM MgCl_2_, 1 mM EDTA, 1 mM dithiothreitol, 1% (v/v) NP‐40 pH 7.5), followed by incubated with GSTSep Glutathione Agarose Resin (YEASAN) for another 2 h. After washing three times, the resins were suspended in 2 × loading buffer and operated with SDS‐PAGE.

### 4.13 RhoA activity assay

The active RhoA in PEMs and RAW264.7 cells was measured with Active Rho Detection Kit (CST) using the manufacturer's instruction.

### 4.14 ChIP assay

PEMs were treated with BSA or UGRP1 (0.5 μg/ml) for 1 h and ChIP assay was operated with rabbit IgG and anti‐p65 antibody. qRT‐PCR was used to quantify the amount of TLR2 and NOD2 DNA fragments from the immunoprecipitated chromatin. Sequences of TLR2 and NOD2 fragments are listed in Table 3.

### 4.15 ELISA

ELISA kits (Invitrogen) were used to measure the concentrations of IL‐6 and TNFα in mouse BAL fluid and cell supernate according to the manufacturer's instruction.

### 4.16 Isolation of nucleic fractions

After treatment with 0.5 μg/ml UGRP1 for the indicated periods, the PEMs were harvested and washed with PBS. Then, buffer A (10 mM HEPES, 1.5 mM MgCl_2_, 10 mM KCl, 0.5 mM dithiothreitol, 0.1% (v/v) NP‐40 and protease inhibitor cocktail, pH 7.9) was used to lyse the cells. After centrifugation at 2152 *g* for 20 min at 4°C, supernate was discarded and the pellet was dissolved with cold buffer C (20 mM HEPES, 1.5 mM MgCl_2_, 0.42 M NaCl, 0.2 mM EDTA, 25% (v/v) glycerol, 0.5 mM dithiothreitol and protease inhibitor cocktail, pH 7.9). Nuclear protein was obtained with centrifugation at 8608 *g* for 1 min at 4°C to remove the insoluble material.

### 4.17 Flow cytometry

Single‐cell suspension was obtained with a 70 μm nylon cell strainer. Then, the living cells were directly stained with the indicated antibodies in PBS containing 5% BSA for surface staining at 4°C in dark for 30 min. For intracellular staining, cells were initially fixed in 1% formaldehyde for 15 min, followed by PBS including 0.1% NP‐40 treatment for 30 min. Then, cells were stained with the indicated antibodies in PBS containing 5% BSA. After washed with cold PBS, Calibur (BD Bioscience) was subsequently used to analyse the samples.

### 4.18 Statistics

All sample sizes were large enough to ensure proper statistical analysis. Data were represented as the means ± SD of at least three experiments. Statistical analyses were performed using GraphPad Prism 8 software (GraphPad Software, Inc.). Statistical significance was calculated using Student's two‐tailed unpaired *t*‐test or analysis of variance with Holm–Sidak's multiple comparisons test. The log‐rank (Mantel–Cox) test was used for survival comparisons: ns, not significant (*p* > .05); *p < .05; **p < .01; ***p < .001.

## CONFLICT OF INTEREST

The authors declare no conflict of interest.

## Supporting information

Supporting Information
