# Supplementary material for: Uterus globulin associated protein 1 (UGRP1) binds podoplanin (PDPN) to promote a novel inflammation pathway during Streptococcus pneumoniae infection
Source: Clin Transl Med. 2022 Jun 2;12(6):e850. doi: 10.1002/ctm2.850 (PMC9161880; doi:10.1002/ctm2.850)
Supplement: Supplementary file 1 — Supporting Information [file CTM2-12-e850-s001.docx]

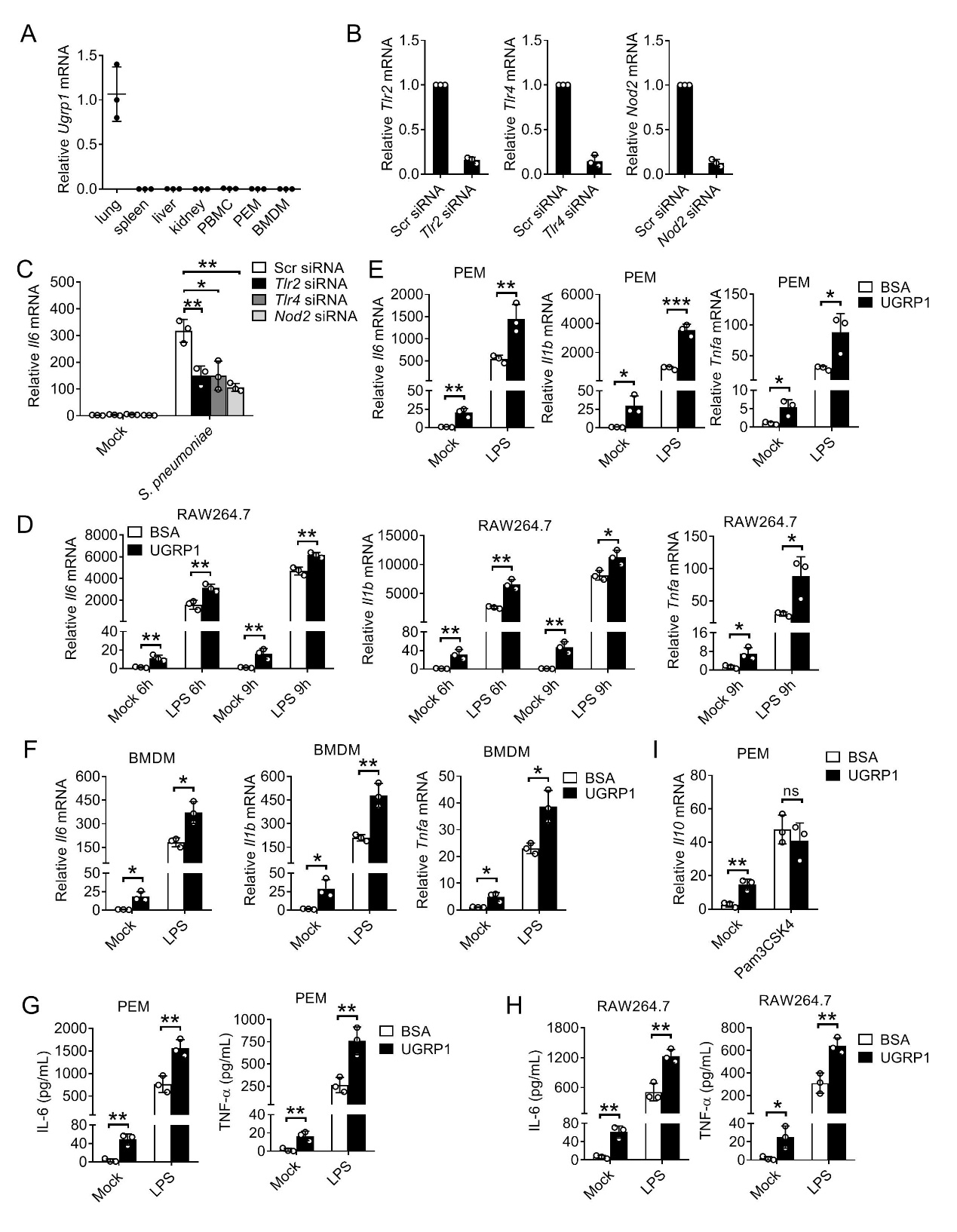


**Figure S1 (related to Figure 1): UGRP1 was identified to positively regulate inflammation in LPS stimulated macrophages**

1. qRT-PCR analysis of *Ugrp1* mRNA in murine lung, spleen, liver, kidney, PBMCs, BMDMs and PEMs (n=3).
2. *Tlr2, Tlr4* and *Nod2* siRNA silencing efficiency in PEMs was confirmed at 48h after transfection.
3. PEMs transfected with *Tlr2, Tlr4* and *Nod2* siRNA for 36h, then treated with *S. pneumoniae* (10^4^cfu/well) for 6h to check *Il6* mRNA by qRT-PCR (n=3).
4. RAW264.7 cells were treated with BSA or UGRP1 (0.5μg/mL) for 1h followed by LPS (0.1 μg/mL) stimulation for 6h or 9h to check *Il6, Il1b* and *Tnfa* mRNA by qRT-PCR (n=3).
5. PEMs were treated with BSA or UGRP1 (0.5μg/mL) for 1h followed by LPS (0.1μg/mL) stimulation for 6h to check *Il6, Il1b* and *Tnfa* mRNA by qRT-PCR (n=3).
6. BMDMs were treated with BSA or UGRP1 (0.5μg/mL) for 1h followed by LPS (0.1μg/mL) stimulation for 6h to check *Il6, Il1b* and *Tnfa* mRNA by qRT-PCR (n=3).

(G-H) PEMs (E, n=3) or RAW264.7 cells (F, n=3) were treated with BSA or UGRP1 (0.5μg/mL) for 1h followed by LPS (0.1μg/mL) stimulation for 12h to check IL-6 and TNFα concentrations by ELISA.

(I) PEMs were treated with BSA or UGRP1 (0.5μg/mL) for 1h followed by Pam3CSK4 (0.5μg/mL) stimulation for 6h to check *Il10* mRNA by qRT-PCR (n=3).

* p<0.05, ** p< 0.01 and *** p<0.001, using one-way ANOVA with Holm-Sidak’s multiple comparisons test (A-B), or two-way ANOVA with Holm-Sidak’s multiple comparisons test (B-I). Data from at least three independent experiments (mean ± SD).


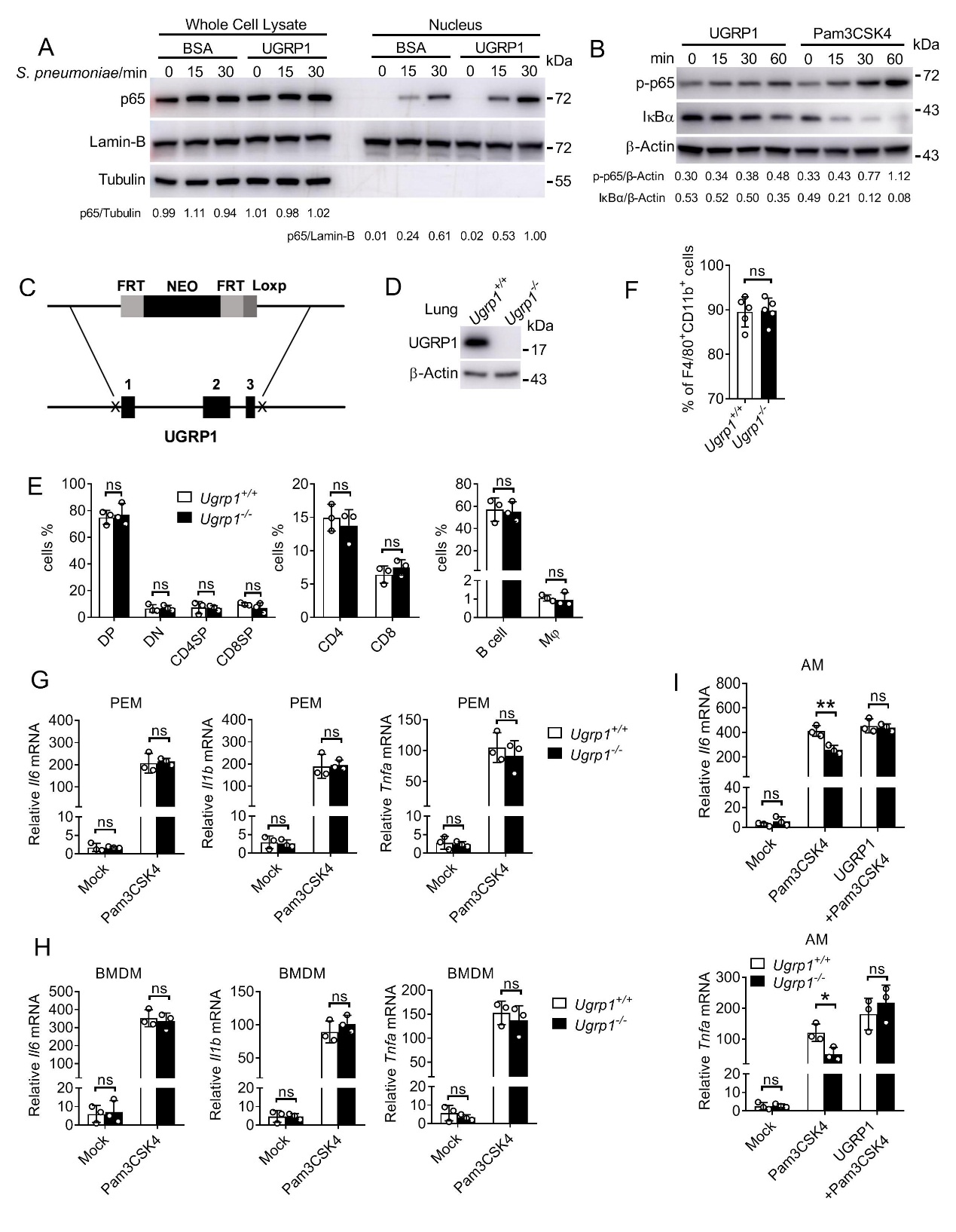


**Figure S2. UGRP1 deficiency did not affect development of lymphocytes.**

(A) Immunoblot analysis p65 in the whole cell lysate (left) and nucleus (right) in BSA or UGRP1 treated PEMs for 6h following *S. pneumoniae* (10^5^cfu/well) stimulation for the indicated periods. Tubulin were used as the whole lysate protein control, Lamin-B served as the nucleic protein control.

(B) PEMs were treated with UGRP1 (0.5μg/mL) or Pam3CSK4 (0.5μg/mL) for indicated periods, p-p65 and degradation of IκBα were analyzed by immunoblot.

(C) Schema of the UGRP1 knockout structure. 3.6kbp genomic sequence including the whole *Ugrp1* gene was replaced by 2.3kbp DNA fragment containing Neomycin cassette.

(D) The efficiency of UGRP1 KO was analyzed by immunoblotting with anti-UGRP1 antibody in lungs from WT and UGRP1 KO mice.

(E) Immune cell subsets in thymus (left panel) or spleens (middle and right panel) from WT or UGRP1 KO mice were analyzed by FACS.

(F) Bone marrow cells were cultured with 30% L929-conditioned media for a week to generate BMDMs. Then, BMDMs were labeled with anti-F4/80 and anti-CD11b antibodies for FACS analysis.

(G-H) PEMs (G) and BMDMs (H) from WT or UGRP1 KO mice were stimulated with Pam3CSK4 (0.5 μg/mL) for 6h to check *Il6, Il1b* and *Tnfa* mRNA by qRT-PCR (n=3).

(I) Alveolar macrophages (AMs) from WT or UGRP1 KO mice were treated with or without UGRP1 for 1h, following stimulation with Pam3CSK4 for 6h to check *Il6* and *Tnfa* mRNA by qRT-PCR (n=3).

* p<0.05, ** p< 0.01 and *** p<0.001, using a two-tailed, unpaired Student’s t test (F) or two-way ANOVA with Holm-Sidak’s multiple comparisons test (E, G, H, I). Data from at least three independent experiments (mean ± SD) or representative data (A, B, D).


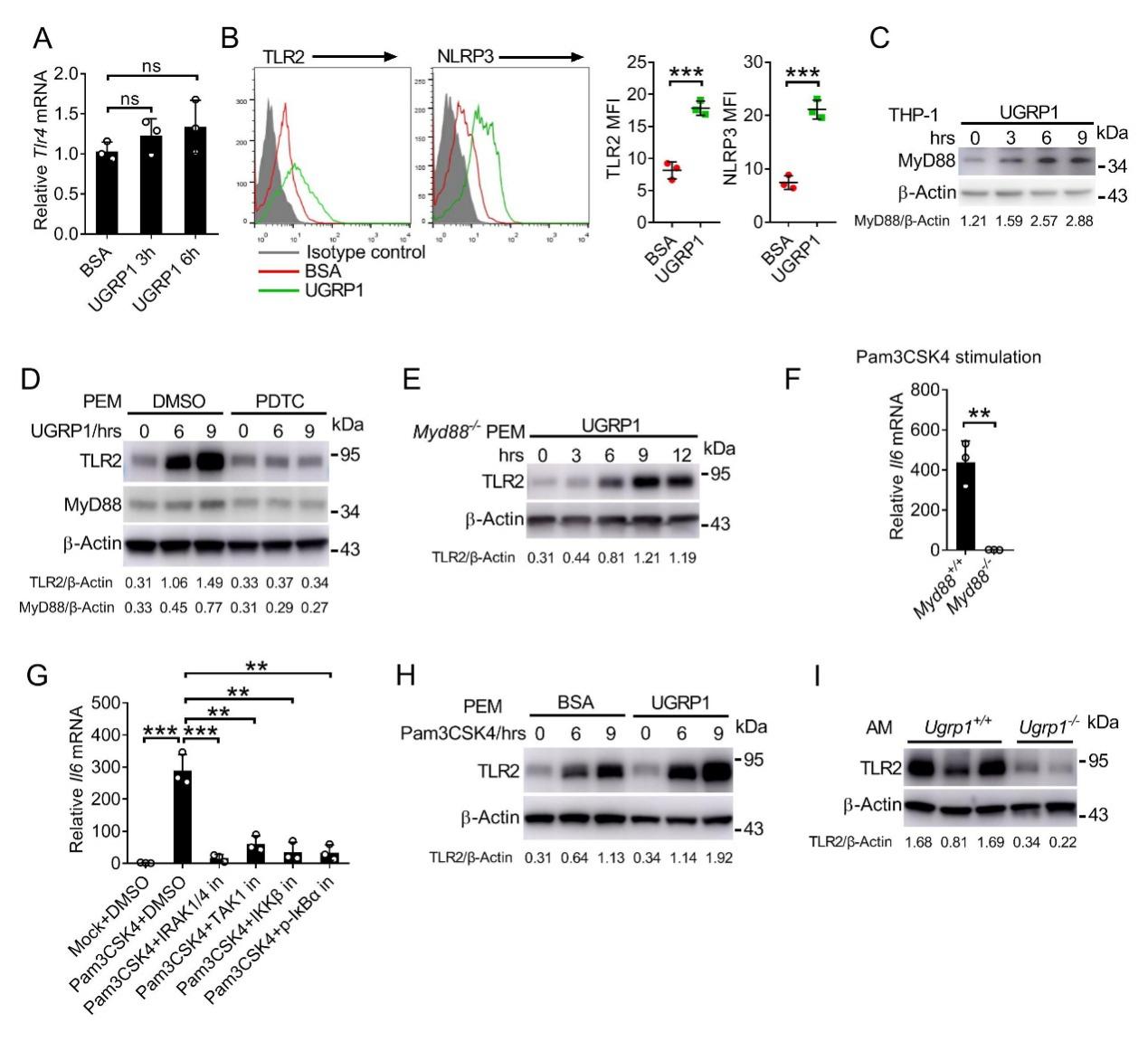


**Figure S3. UGRP1 enhances expression of TLR2, NOD2, MyD88 and NLRP3 by activation of NF-κB.**

(A) PEMs were treated with UGRP1 (0.5 μg/mL) for 3h or 6h to check *Tlr4* mRNA by qRT-PCR (n=3).

(B) PEMs were treated with UGRP1 (0.5 μg/mL) for 9h to measure TLR2 protein levels in cell surface and NLRP3 expression by FACS analysis (n=3).

(C) PMA-induced human THP-1 cells were treated with UGRP1 (0.5μg/mL) for indicated periods to measure MyD88 protein levels by immunoblot analysis.

(D) PEMs were treated with the NF-κB inhibitor PDTC (100μM) for 1h followed by BSA or UGRP1 (0.5μg/mL) treatment for 6h or 9h to measure TLR2 and MyD88 protein levels by immunoblot analysis.

(E) MyD88 KO PEMs were treated with UGRP1 (0.5 μg/mL) for indicated periods to measure TLR2 protein levels by immunoblot analysis.

(F) WT and MyD88 KO PEMs were stimulated with Pam3CSK4 (0.5μg/mL) for 6h to measure *Il6* mRNA by qRT-PCR (n=3).

(G) PEMs were treated with IRAK1/4 inhibitor IRAK-1-4 Inhibitor I (10μM), TAK1 inhibitor Takinib (10μM), IKKβ inhibitor LY2409881 trihydrochloride (5μM) or p-IκBα inhibitor BAY 11-7082 (20μM) for 1h followed by Pam3CSK4 (0.5μg/mL) stimulation for 6h to measure *Il6* mRNA by qRT-PCR (n=3).

(H) PEMs were stimulated by Pam3CSK4 (0.5μg/mL) with BSA or UGRP1 (0.5μg/mL) for 6h or 9h to measure TLR2 protein levels by immunoblot analysis.

(I) Immunoblot analysis of TLR2 in AMs from WT and UGRP1 KO mice.

* p<0.05, ** p< 0.01 and *** p<0.001, using a two-tailed, unpaired Student’s t test (B right panel, F) or one-way ANOVA with Holm-Sidak’s multiple comparisons test (A, G). Data from at least three independent experiments (mean ± SD) or representative data (B left panel, C, D, E, H, I).


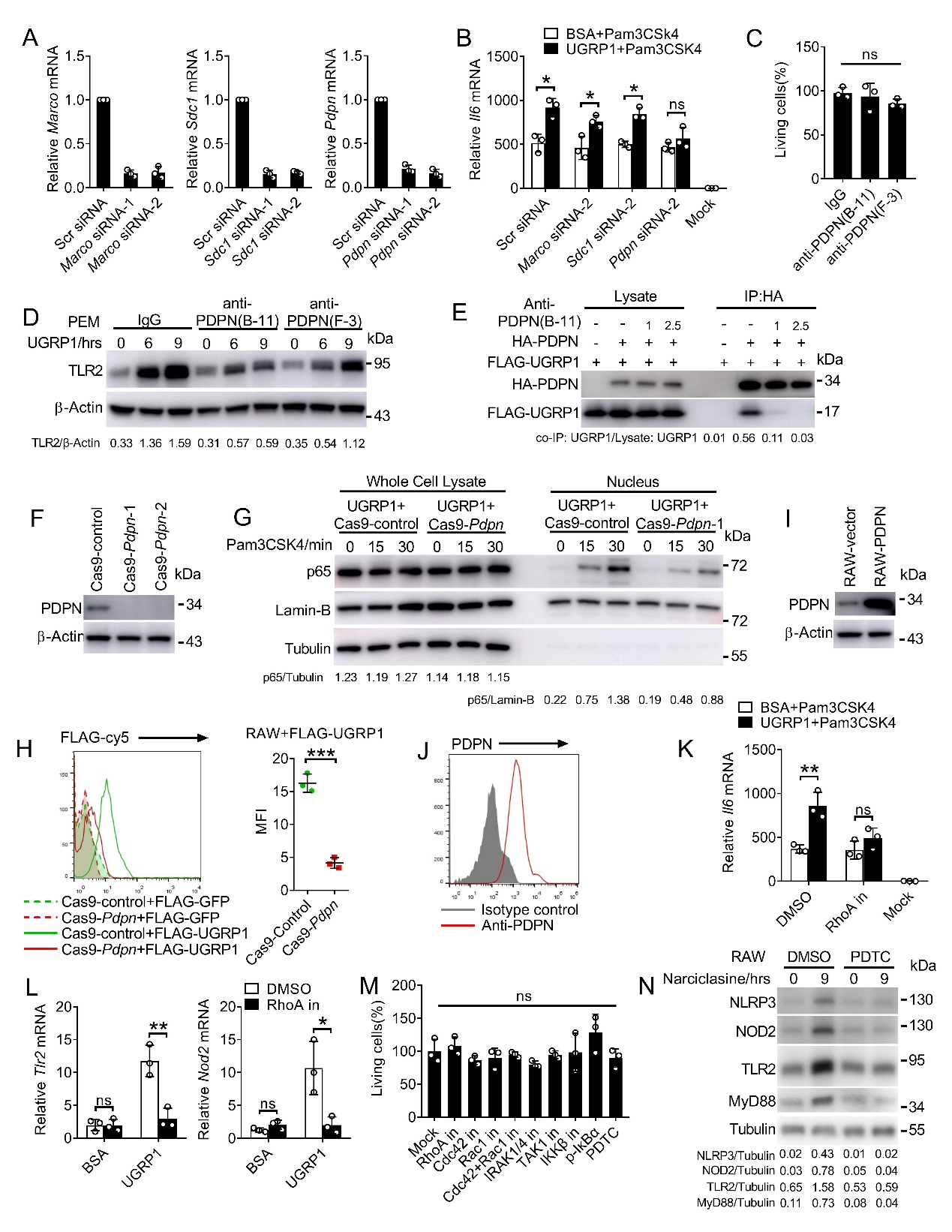


**Figure S4. UGRP1-PDPN signaling activates RhoA to regulate TLR2-induced inflammation.**

(A) *Marco, Sdc1* and *Pdpn* siRNA silencing efficiency in PEMs was confirmed at 48h after transfection.

(B) PEMs were transfected with control siRNA, *Marco* siRNA, *Sdc1* siRNA or *Pdpn* siRNA followed by Pam3CSK4 (0.5μg/mL) stimulation for 6h with BSA or UGRP1 (0.5μg/mL) treatment and the *Il6* mRNA levels were checked using qRT-PCR (n=3).

(C) PEMs were treated control IgG or two PDPN antibodies (2μg/mL) named anti-PDPN (B11) or anti-PDPN (F3) for 12h followed by CCK-8 assay (n=3).

(D) PEMs were incubated with control IgG or two PDPN antibodies (2μg/mL) named anti-PDPN (B11) or anti-PDPN (F3) for 1h followed by UGRP1 treatment for indicated periods to check TLR2 expression by immunoblot assay

(E) HA-Vector and HA-tagged PDPN were transfected into 293T cells respectively with FLAG-tagged UGRP1. Then, B-11 antibody (1 or 2.5μg/mL) was added to the 293T medium for 36h. Immunoprecipitation and immunoblot were performed with the indicated antibodies.

(F) Immunoblot assay of PDPN in Cas9-*Pdpn-*deficient RAW264.7 cells.

(G) Cas9-control and Cas9-*Pdpn*-deficient RAW264.7 cells were treated with UGRP1 (0.5μg/mL) for 6h followed by Pam3CSK4 (0.5μg/mL) stimulation for the indicated periods. p65 in the whole cell lysate and nucleus was analyzed by immunoblot.

(H) Cas9-control and Cas9-*Pdpn*-deficient RAW264.7 cells were incubated with purified FLAG-GFP or FLAG-UGRP1 (0.5μg/mL) for 0.5h followed by anti-FLAG antibody (2μg/mL) incubation for 0.5h then stained with Cy5 conjugated anti-mouse IgG antibody. The cells were analyzed by flow cytometry.

(I) Immunoblot assay of PDPN-overexpressed RAW264.7 cells.

(J) Flow cytometry of cell surface PDPN expression in AMs

(K) PEMs were treated with DMSO control or RhoA inhibitor (CCG-1423, 20μM) for 1h followed by UGRP1 (0.5μg/mL) and Pam3CSK4 (0.5μg/mL) stimulation for 6h to measure *Il6* mRNA levels by qRT-PCR (n=3).

(L) PEMs were treated with DMSO or RhoA inhibitor (CCG-1423, 20μM) for 1h followed by UGRP1 (0.5μg/mL) incubation for 6h to measure *Tlr2* and *Nod2* mRNA levels by qRT-PCR (n=3).

(M) PEMs were treated with RhoA inhibitor (CCG-1423, 20μM), Cdc42 inhibitor (CASIN, 20μM), Rac1 inhibitor (1A-116, 10μM), dual Cdc42/Rac1 inhibitor (MBQ-167, 10μM), IRAK1/4 inhibitor IRAK-1-4 Inhibitor I (10μM), TAK1 inhibitor Takinib (10μM), IKKβ inhibitor LY2409881 trihydrochloride (5μM), p-IκBα inhibitor BAY 11-7082 (20μM) or NF-κB inhibitor PDTC (100μM) for 10h followed by CCK-8 assay (n=3).

(N) RAW264.7 cells were treated with DMSO control or NF-κB inhibitor PDTC (100μM) for 1h followed by RhoA activator (Narciclasine, 0.1μM) for 9h to measure NLRP3, NOD2, TLR2 and MyD88 expression by immunoblot analysis.* p<0.05, ** p< 0.01 and *** p<0.001, using one-way ANOVA with Holm-Sidak’s multiple comparisons test (A, C, H right panel, M) or two-way ANOVA with Holm-Sidak’s multiple comparisons test (B, K, L). Data from at least three independent experiments (mean ± SD) or representative data (D, E, F, G, H left panel, I, J, N).


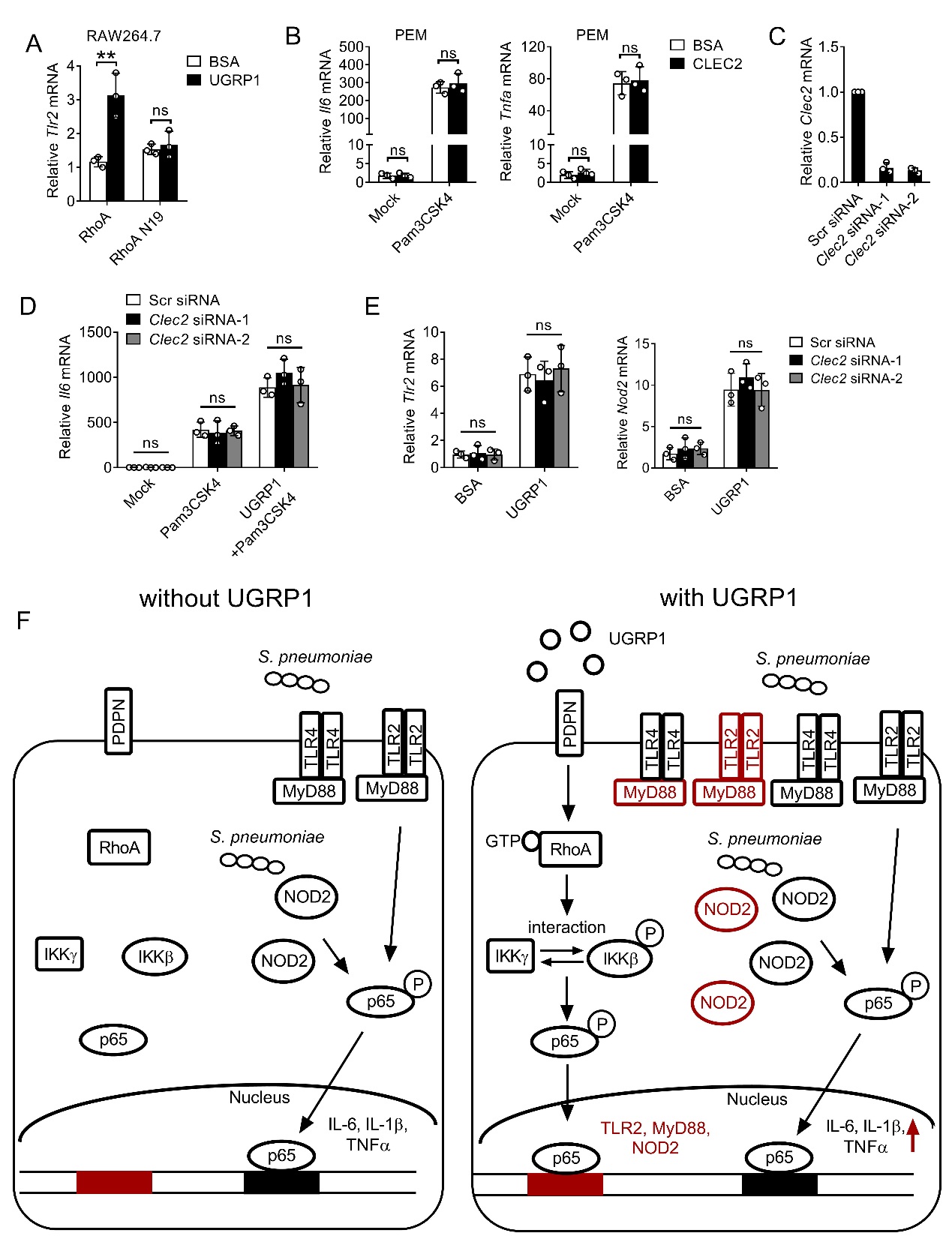


**Figure S5. UGRP1 activates RhoA to enhance the interaction of IKKγ and IKKβ.**

(A) RAW264.7 cells stably overexpressing WT RhoA or RhoA N19 mutant were treated with BSA or UGRP1 (0.5μg/mL) for 6h to measure *Tlr2* mRNA by qRT-PCR (n=3).

(B) PEMs were treated with BSA or CLEC-2 (0.5μg/mL) for 1h followed by Pam3CSK4 (0.5μg/mL) for 6h to check *Il6* and *Tnfa* mRNA by qRT-PCR (n=3).

(C) *Clec2* siRNA silencing efficiency in PEMs was confirmed at 48h after transfection.

(D) PEMs were transfected with scrambled siRNA or *Clec2* siRNA followed by Pam3CSK4 (0.5μg/mL) with or without UGRP1 (0.5μg/mL) stimulation for 6h and the *Il6* mRNA levels were checked using qRT-PCR (n=3).

(E) PEMs were transfected with scrambled siRNA or *Clec2* siRNA followed by UGRP1 (0.5μg/mL) stimulation for 6h and the Tlr2 and Nod2 mRNA levels were checked using qRT-PCR (n=3).

(F) UGRP1-PDPN signaling activate RhoA to enhance the interaction of IKKγ and IKKβ, which slightly activate NF-κB to enhance expression of TLR2, MyD88 and NOD2. Upon *S. pneumoniae* infection, increased expression of TLR2, MyD88 and NOD2 could generate enhanced NF-κB signaling to produce more pro-inflammatory cytokines including IL-6, IL-1β and TNFα.

* p<0.05, ** p< 0.01 and *** p<0.001, using one-way ANOVA with Holm-Sidak’s multiple comparisons test (C) or two-way ANOVA with Holm-Sidak’s multiple comparisons test (A, B, D, E).


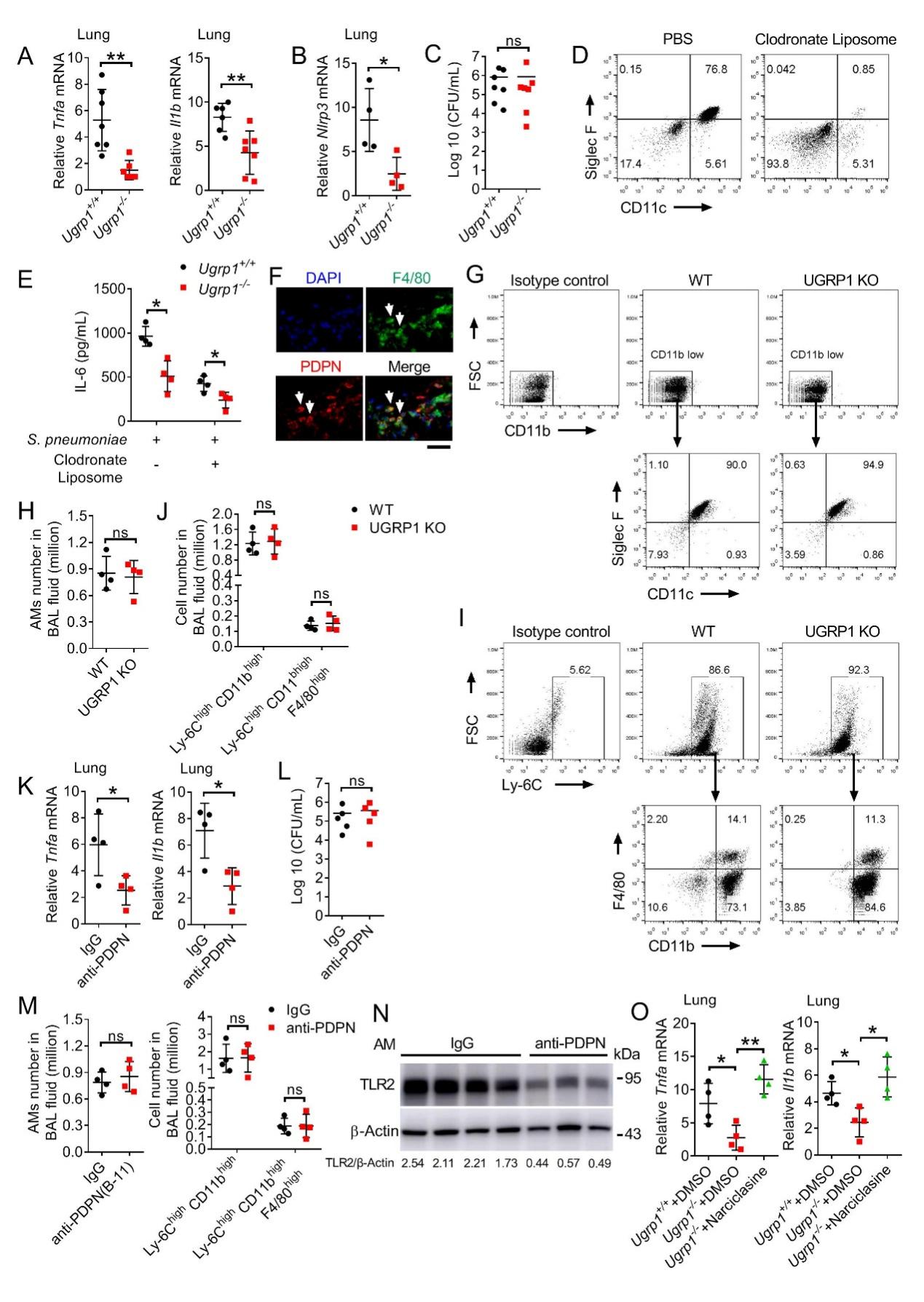


**Figure S6. UGRP1 deficiency protects against *S. pneumoniae*-induced pneumonia.**

(A-C) WT and UGRP1 KO mice were infected intratracheally with *S. pneumoniae* (10^5^ cfu/mouse). Lungs were harvested at 1 day post infection and *Tnfa*, *Il1b* (A, n=6) and *Nlrp3* (B, n=4) mRNA levels were checked by qRT-PCR. BAL fluid was prepared at 1 day after inoculation and *S. pneumoniae* counts were determined (C, n=7).

(D) WT and UGRP1 KO mice were intratracheally injected with Clodronate Liposomes (0.25mg/mouse). 2 days later, BAL fluid was collected and AMs (CD11c^high^ Siglec F^high^ CD11b^low^) were analyzed by flow cytometry.

(E) WT and UGRP1 KO mice were intratracheally injected with Clodronate Liposomes (0.25mg/mouse) for 2 days and infected intratracheally with *S. pneumoniae* (10^5^ cfu/mouse). IL-6 in BAL fluid was detected by ELISA at 1 day after inoculation (n=4).

(F) WT mice were infected intratracheally with *S. pneumoniae* (10^5^ cfu/mouse). 1 day later, staining of lung tissue was performed with anti-F4/80 and anti-PDPN (B-11) (scale bar: 20μm).

(G-H) BAL fluid from WT and UGRP1 KO mice was prepared and AMs (CD11c^hi^ Siglec F^hi^ CD11b^low^) subsets were analyzed by FACS (G). The number of AMs was counted (H, n=4).

(I-J) WT and UGRP1 KO mice were injected intratracheally with PBS or *S. pneumoniae* (10^5^ cfu/mouse). BAL fluid was prepared at 1 day after inoculation. Infiltrated monocytes (Ly-6C^high^ CD11b^high^) and macrophages (Ly-6C^high^ CD11b^high^ F4/80^high^) subsets were analyzed by FACS (I). The number of infiltrated monocytes and macrophages was counted (J, n=4)

(K-L) WT mice pre-treated with control IgG or anti-PDPN (B-11) antibody (1μg/mouse) were infected intratracheally with *S. pneumoniae* (10^5^ cfu/mouse). Lungs were harvested at 1 day post infection and *Tnfa* and *Il1b* mRNA levels were checked by qRT-PCR (K, n=4). BAL fluid was prepared at 1 day after inoculation and *S. pneumoniae* counts were determined (L, n=5).

(M) BAL fluid from WT mice pre-treated with control IgG or anti-PDPN (B-11) antibody (1μg/mouse) was prepared and AMs (CD11c^hi^ Siglec F^hi^ CD11b^low^) number was counted (left, n=4). WT pre-treated with control IgG or anti-PDPN (B-11) antibody (1μg/mouse) were injected intratracheally with PBS or *S. pneumoniae* (10^5^ cfu/mouse). BAL fluid was prepared at 1 day after inoculation. Infiltrated monocytes (Ly-6C^high^ CD11b^high^) and macrophages (Ly-6C^high^ CD11b^high^ F4/80^high^) number was counted (right, n=4)

(N) Immunoblot analysis of TLR2 in AMs from WT pre-treated with control IgG or anti-PDPN (B-11) antibody (1μg/mouse) for 2 days.

(O) WT and UGRP1 KO mice pre-treated with DMSO or RhoA activator (Narciclasine, 5μg/mouse) were infected intratracheally with *S. pneumoniae* (10^5^ cfu/mouse). Lungs were harvested at 1 day post infection and *Tnfa* and *Il1b* mRNA levels were checked by qRT-PCR (n=4).

* p<0.05, ** p< 0.01 and *** p<0.001, using a two-tailed, unpaired Student’s t test (A, B, C, H, K, L, M left panel), one-way ANOVA with Holm-Sidak’s multiple comparisons test (O) or two-way ANOVA with Holm-Sidak’s multiple comparisons test (E, J, M right panel). Data from at least three independent experiments (mean ± SD) or representative data (D, G, I, N).
